# Respiratory protein-driven selectivity during the Permian-Triassic mass extinction

**DOI:** 10.1016/j.xinn.2024.100618

**Published:** 2024-03-28

**Authors:** Haijun Song, Yuyang Wu, Xu Dai, Jacopo Dal Corso, Fengyu Wang, Yan Feng, Daoliang Chu, Li Tian, Huyue Song, William J. Foster

**Affiliations:** 1 State Key Laboratory of Biogeology and Environmental Geology, School of Earth Sciences, China University of Geosciences, Wuhan 430074, China; 2 Biogéosciences, UMR 6282, CNRS, Université de Bourgogne, 21000 Dijon, France; 3 Universität Hamburg, Institute for Geology, 20148 Hamburg, Germany

## Abstract

Extinction selectivity determines the direction of macroevolution, especially during mass extinction; however, its driving mechanisms remain poorly understood. By investigating the physiological selectivity of marine animals during the Permian-Triassic mass extinction, we found that marine clades with lower O_2_-carrying capacity hemerythrin proteins and those relying on O_2_ diffusion experienced significantly greater extinction intensity and body-size reduction than those with higher O_2_-carrying capacity hemoglobin or hemocyanin proteins. Our findings suggest that animals with high O_2_-carrying capacity obtained the necessary O_2_ even under hypoxia and compensated for the increased energy requirements caused by ocean acidification, which enabled their survival during the Permian-Triassic mass extinction. Thus, high O_2_-carrying capacity may have been crucial for the transition from the Paleozoic to the Modern Evolutionary Fauna.

## Public summary

• Physiological traits were key in determining species’ survival during the Permian-Triassic mass extinction.
• Clades with hemoglobin and hemocyanin exhibited high survival rates during the mass extinction.
• Clades with lower O_2_-carrying capacity experienced a significant reduction in body size during anoxic events.

## Graphical abstract

**Figure:**
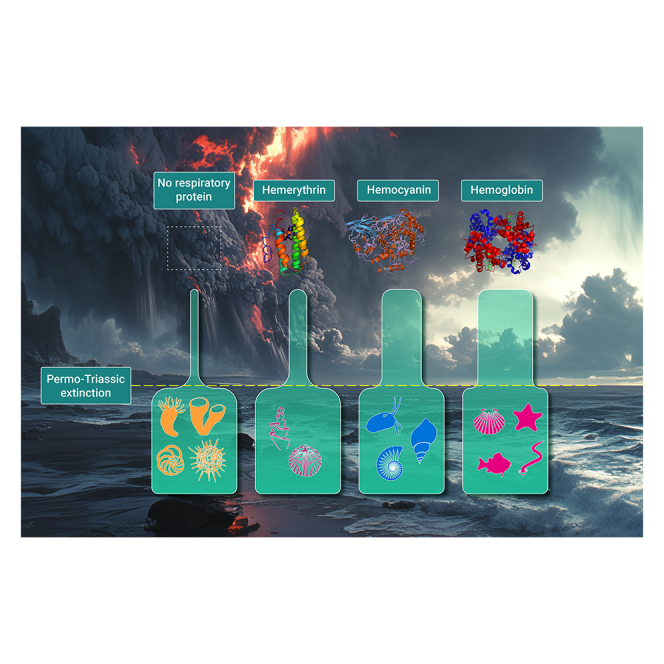

## Introduction

In the Phanerozoic, the rates of extinction were particularly high during short intervals of time, called the “Big Five” mass extinctions.^1^^,^^2^ Despite the high magnitude of biological loss observed during these events, extinction did not affect all groups with the same intensity; that is, some groups experienced high extinction rates or completely disappeared, whereas others survived without similar losses.^3^^,^^4^ This extinction selectivity determined the patterns of macroevolution following biological crises^5^^,^^6^; however, why extinctions were selective across clades remains poorly understood.

The Permian-Triassic mass extinction was the most severe biological crisis of the Phanerozoic, with a loss of over 80% of marine species,^7^^,^^8^ and it determined the pivotal transition in the history of life from the Paleozoic to the Modern Evolutionary Fauna,^9^^,^^10^ which has also been called Mesozoic and Cenozoic communities and has more diverse predators and more complex predator-prey interactions.^11^^,^^12^ Extinction selectivity based on ecological and phylogenetic criteria has been observed in the Permian-Triassic fossil record.^3^^,^^4^^,^^13^^,^^14^^,^^15^ For example, organisms with a heavy carbonate load and limited circulatory system were preferentially removed, interpreted as a consequence of elevated *p*CO_2_ and hypercapnia.^3^^,^^13^^,^^14^ Body-size selectivity was also observed during the Permian-Triassic mass extinction: the survivors of some groups, such as foraminifera and brachiopods, were significantly smaller, whereas others (e.g., ammonoids and fish) showed little change in body size.^16^^,^^17^^,^^18^^,^^19^

**Figure 1:**
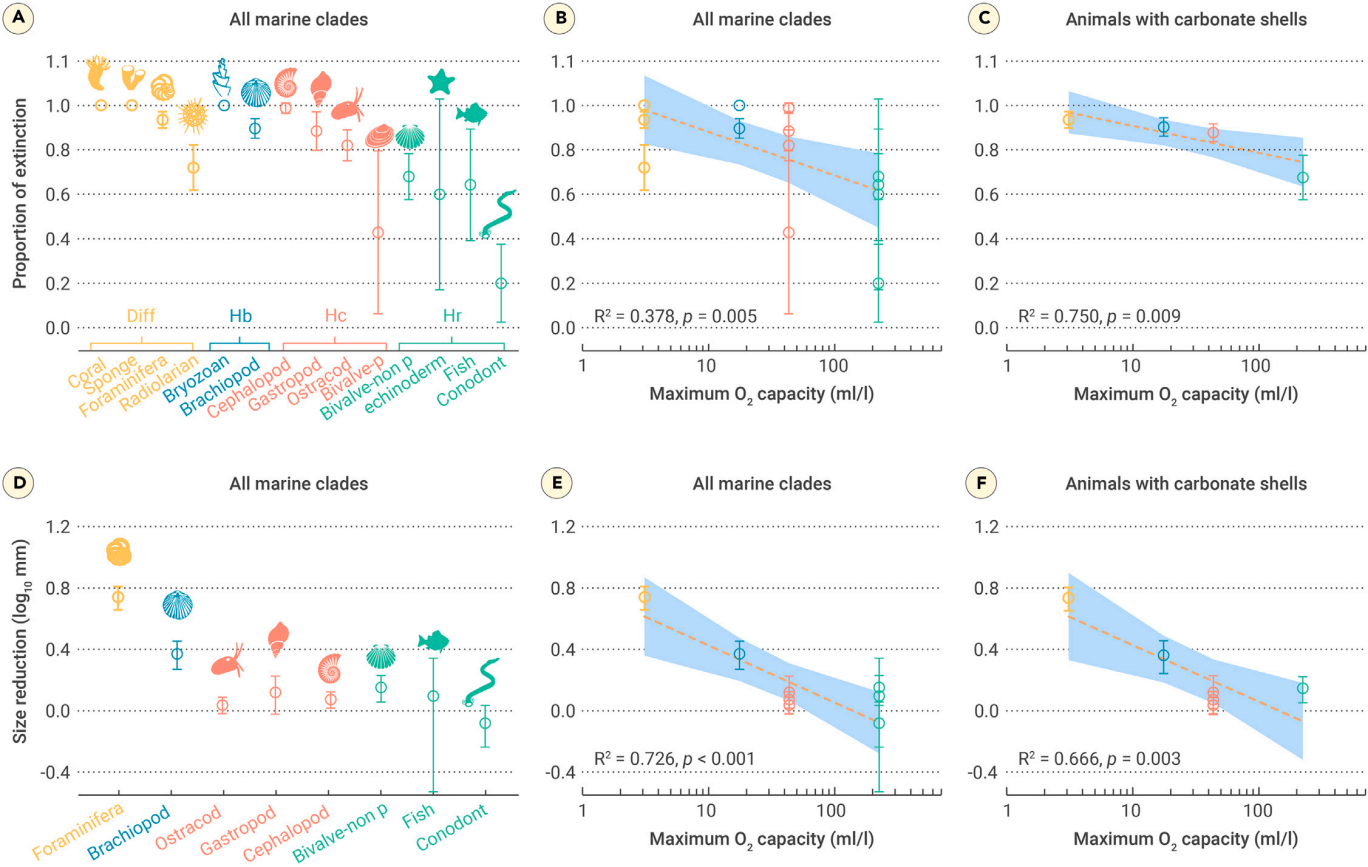
Extinction and body-size reduction of marine animals at the genus level during the Permian-Triassic mass extinction (A) Extinction magnitudes of major marine clades. (B) Correlation between the proportion of extinction and O_2_ capacity for marine clades. (C) Correlation between the proportion of extinction and O_2_ capacity for animals with carbonate shells. (D) Size reduction of major marine clades. (E) Correlation between size reduction and O_2_ capacity for marine clades. (F) Correlation between size reduction and O_2_ capacity for animals with carbonate shells. Vertical bars represent binomial 95% confidence intervals for (A)–(C). Vertical bars in (D)–(F) represent the standard deviation, which was calculated from 1,000 bootstrap replicates of median size reduction. Diff (orange color), Hr (purple color), Hc (blue color), and Hb (magenta color) represent different types of animals that use diffusion, hemerythrin, hemocyanin, and hemoglobin, respectively, to transport O_2_ from the surroundings to their bodies. Bivalve-p and bivalve-non p represent protobranchia bivalve and hemoglobin non-protobranchia bivalve, respectively. Dashed lines and shades represent linear regression lines and their 95% confidence intervals. For the methods used to calculate extinction and size reduction and their error bars, see the supplemental materials and methods.

Species with narrow geographic ranges are generally thought to be more likely to become extinct^20^^,^^21^; however, geographic range is not a strong predictor of selectivity for mass extinctions.^13^^,^^21^ Skeletal composition, which is considered a good predictor of extinction risk,^13^ cannot explain selectivity in clades with the same mineralogical composition (such as carbonate shells) with respect to extinction and size reduction, as observed in the Permian-Triassic transition (Figure 1C).

The paleophysiology of marine animals can provide important insights into the mechanisms of extinction selectivity,^3^ but it often lacks independent and quantifiable indicators. For example, physiological buffering capacity, which is the most commonly used paleophysiological indicator, is inferred from skeletal mineralogy and both respiratory and circulatory anatomy.^3^^,^^15^ In the present study, we directly considered a physiological attribute of marine animals—that is, respiratory proteins—using quantifiable O_2_-carrying capacity to investigate its role in driving the selective extinction and body-size reduction observed during the Permian-Triassic crisis.

Respiratory proteins are important for controlling physiological activities, including O_2_ transportation, respiration, and energy supply.^22^^,^^23^ Permian-Triassic marine animals can be classified into four respiratory protein groups according to their O_2_-carrying types, namely diffusion (without respiratory protein), hemerythrin, hemocyanin, and hemoglobin (Table S1), based on reference protein data for modern animals and assuming that extinct organisms had the same protein type and O_2_-carrying capacity as modern organisms of the same clade^23^ (Table S2). The diffusion group consists of protozoa, sponges, and corals, which do not have O_2_-carrying proteins in their bodies and rely solely on O_2_ diffusion in seawater to transport O_2_ for respiration. The other three groups consist of organisms that possess O_2_-transfer proteins, including hemerythrin (brachiopods and bryozoans), hemocyanin (ostracods, gastropods, cephalopods, and protobranchia bivalves), and hemoglobin (non-protobranchia bivalves, echinoderms, conodonts, and fish). These respiratory proteins have distinct O_2_-carrying capacities^23^ (Table S1). Animals with hemerythrin have a lower O_2_-carrying capacity than those with hemocyanin and hemoglobin, as the hemerythrin concentration in their coelomic fluid and the Hill coefficient (the cooperativity of ligand binding) are substantially lower than those of hemocyanin and hemoglobin. We show that O_2_-carrying capacity played a key role in extinction selectivity during the Permian-Triassic transition, favoring the preferential survival of animals with hemoglobin and hemocyanin, which subsequently dominated the Mesozoic Evolutionary Fauna.

## Results

### Selectivity in extinction

Fossil data used to calculate extinction were updated from an existing database of Permian-Triassic marine fossils.^24^ We used 1,097 genera belonging to 13 major clades in the Changhsingian and Induan stages. Our analysis showed that extinction was most pronounced for marine animals assumed to have had low O_2_-carrying capacity, that is, those that used diffusion or hemerythrin to transport O_2_ (Figure 1A). Corals and sponges were the most severely affected during the Permian-Triassic mass extinction, with the complete extinction of rugose and tabulate corals and more than 90% extinction of the sponge genera. Similarly, the extinction rate of foraminifera, brachiopods, and bryozoans was high (94.4% on average). Radiolarians suffered less extinction (72.0%) than the other animals in the diffusion group. Marine clades possessing hemocyanin or hemoglobin suffered less extinction (65.6% on average) than most clades possessing diffusion or hemerythrin (Figure 1A). Per-capita, three-timer, and gap-filler extinction estimators (which have their own strengths in dealing with sampling biases) showed a significant negative relationship between O_2_-carrying capacity and extinction magnitude for the four fossil groups (Figures S1 and S2).

**Figure 2:**
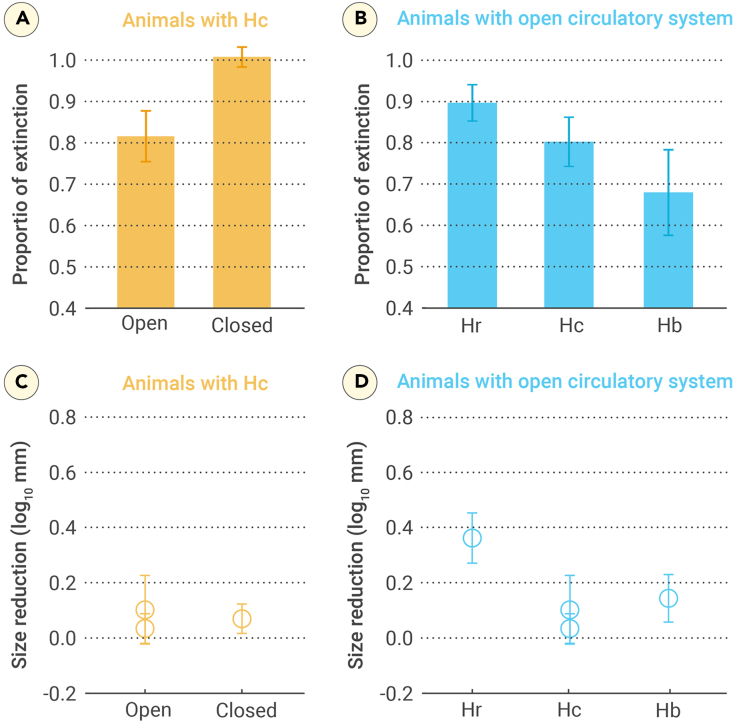
Extinction and size reduction in different groups (A) Differences in extinction between animals with open and closed circulatory systems in the Hc group. The open circulatory system bin includes brachiopods, ostracods, gastropods, and non-protobranchia bivalves; the closed circulatory system bin includes cephalopods. (B) Differences in extinction between animals with three O_2_-carrying proteins in the open circulatory system group. The Hr bin includes brachiopods; the Hc bin includes ostracods, gastropods, and protobranchia bivalves; and the Hb bin includes non-protobranchia bivalves. (C) Differences in size reduction between animals with open and closed circulatory systems in the Hc group. The open circulatory system bin includes ostracods and gastropods; the closed circulatory system bin includes cephalopods. (D) Differences in size reduction between animals with three O_2_-carrying proteins in the open circulatory system group. The Hr bin includes brachiopods; the Hc bin includes ostracods and gastropods; and the Hb bin includes non-protobranchia bivalves. Vertical bars represent the 95% confidence intervals for (A) and (B) and standard deviation for (C) and (D). Hr, Hc, and Hb represent hemoglobin, hemocyanin, and hemoglobin, respectively.

Furthermore, we observed a significant negative correlation between O_2_-carrying capacity and extinction (R^2^ = 0.378, *p* = 0.005; Figure 1B). This relationship was still significant when we excluded the effects of differences in skeletal composition (Figure 1C). The extinction of animals with carbonate skeletons decreased significantly with increasing O_2_-carrying capacity (R^2^ = 0.750, *p* = 0.009; Figure 1C), with extinction magnitudes of 93.5% for diffusion types, 90.2% for animals with hemerythrin, and 87.7% and 67.5% for animals with hemocyanin and hemoglobin, respectively. For animals with hemocyanin, the extinction proportion was substantially higher in the closed circulatory system bin than in the open circulatory system bin (Figure 2A). In animals with open circulatory systems, the extinction magnitude decreased with the decreasing O_2_-carrying capacity from hemerythrin to hemoglobin bins (Figure 2B).

**Figure 3:**
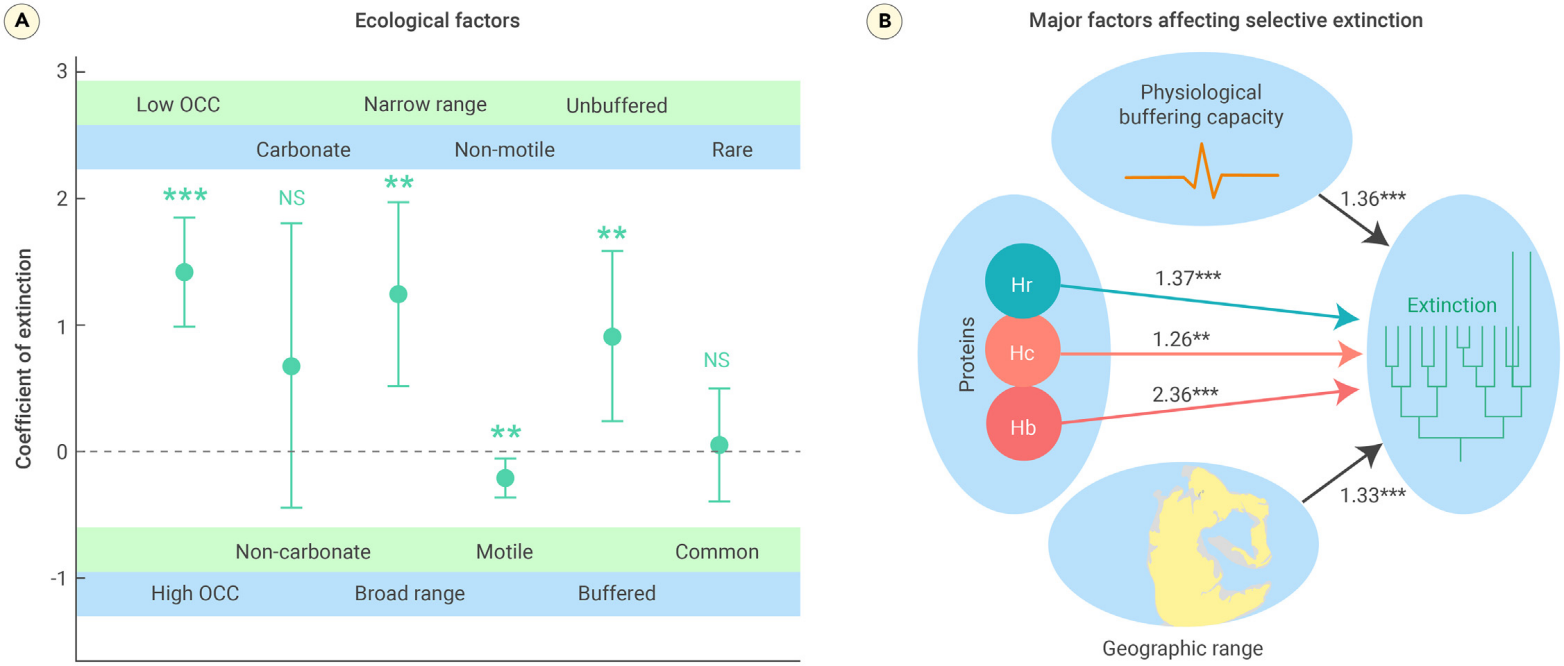
Logistic regression shows the selectivity of extinction during the Permian-Triassic crisis (A) Regression coefficients of extinction. Low O_2_-carrying capacity, narrow geographic range, motile, and physiologically unbuffered genera preferentially went extinct. Selectivity among genera with carbonate shells and a smaller number of occurrences was not significant. (B) Regression coefficients of extinction with O_2_-carrying capacity as categorical covariates. OCC, O_2_-carrying capacity. Vertical bars in (A) represent the standard errors of the regression coefficients. ∗∗∗*p* < 0.001 and ∗∗*p* < 0.01, NS, not significant. For the predictor variables and extinction status, see Table S3, and detailed results of logistic regression are shown in Tables S4–S5.

The results of the multiple logistic regression analyses indicated a highly significant correlation between extinction and O_2_-carrying capacity (*p* < 0.001; Figure 3A; Tables S3–S9). We also found a notable correlation between extinction and geographic range (Figure 3). In addition, the buffering capacity of organisms played a significant role in extinction (Figure 3B); however, the relationship between the number of occurrences and extinction was not statistically significant (*p* = 0.802). Multiple logistic regression with O_2_-carrying capacity as a categorical covariate showed that hemoglobin was the variable with the highest coefficient of extinction selectivity (Figure 3B).

### Selectivity in body size

Size data (expressed as the maximum length for each taxon) were compiled from several recently published datasets and taxonomic literature (see materials and methods). Using the maximum size per taxon is a common approach for body-size studies, as the effects of juvenile specimens can be avoided.^18^^,^^25^ The Changhsingian and Induan body-size datasets comprised 1,495 species in 635 genera belonging to eight common clades. The differences in size variation among the major clades during mass extinction were notable (Figures 1 and S3‒S4), and we observed a significant negative relationship between size reduction and O_2_-carrying capacity (R^2^ = 0.726, *p* < 0.001; Figure 1E). This correlation was still evident when the effects of differences in shell composition were excluded (R^2^ = 0.666, *p* = 0.003; Figures 1F and S4). Foraminifera and brachiopods showed significant reductions in shell length during the Permian-Triassic extinction (Mann-Whitney U test, *p* < 0.001). In contrast, the other clades showed only a weak reduction in body size (ostracods, Mann-Whitney U test, *p* = 0.012) or insignificant size changes (gastropods, cephalopods, bivalves, conodonts, and fish, Mann-Whitney U test, *p* > 0.05; Figure 1D).

Body size was reduced weakly in carbonate-shell animals that used hemocyanin and hemoglobin but remarkably reduced in carbonate animals relying on diffusion and hemerythrin (Figure 1F). For animals with hemocyanin, we observed no marked difference in size reduction between open and closed circulatory system bins (Figure 2C). However, for animals with open circulatory systems, the size reduction was substantially higher in the hemerythrin bin than in the hemocyanin and hemoglobin bins (Figure 2D).

## Discussion

### Mechanisms of selectivity in extinction and size reduction

Our results show that marine animals with hemoglobin and hemocyanin had markedly better resistance to the Permian-Triassic boundary environmental changes than those with hemerythrin or diffusion and both their diversity and body size were significantly less affected (Figure 1). The significant correlation between respiratory proteins and extinction persisted even after controlling for geographic range and skeletal minerology (Figure 3A); this suggests that O_2_-carrying capacity is a key determinant of extinction risk in the marine realm. One factor that might have made respiratory protein types critical for survival during the Permian-Triassic mass extinctions is that respiratory proteins are coupled to the evolution of the respiratory and circulatory systems.^26^ Higher levels of O_2_-carrying proteins generally correspond to advanced circulatory and respiratory systems.^23^^,^^27^^,^^28^ However, when investigating the significance of circulatory systems, we found that repository protein remained a more critical predictor of selectivity in extinction and size reduction (Figure 2).

**Figure 4:**
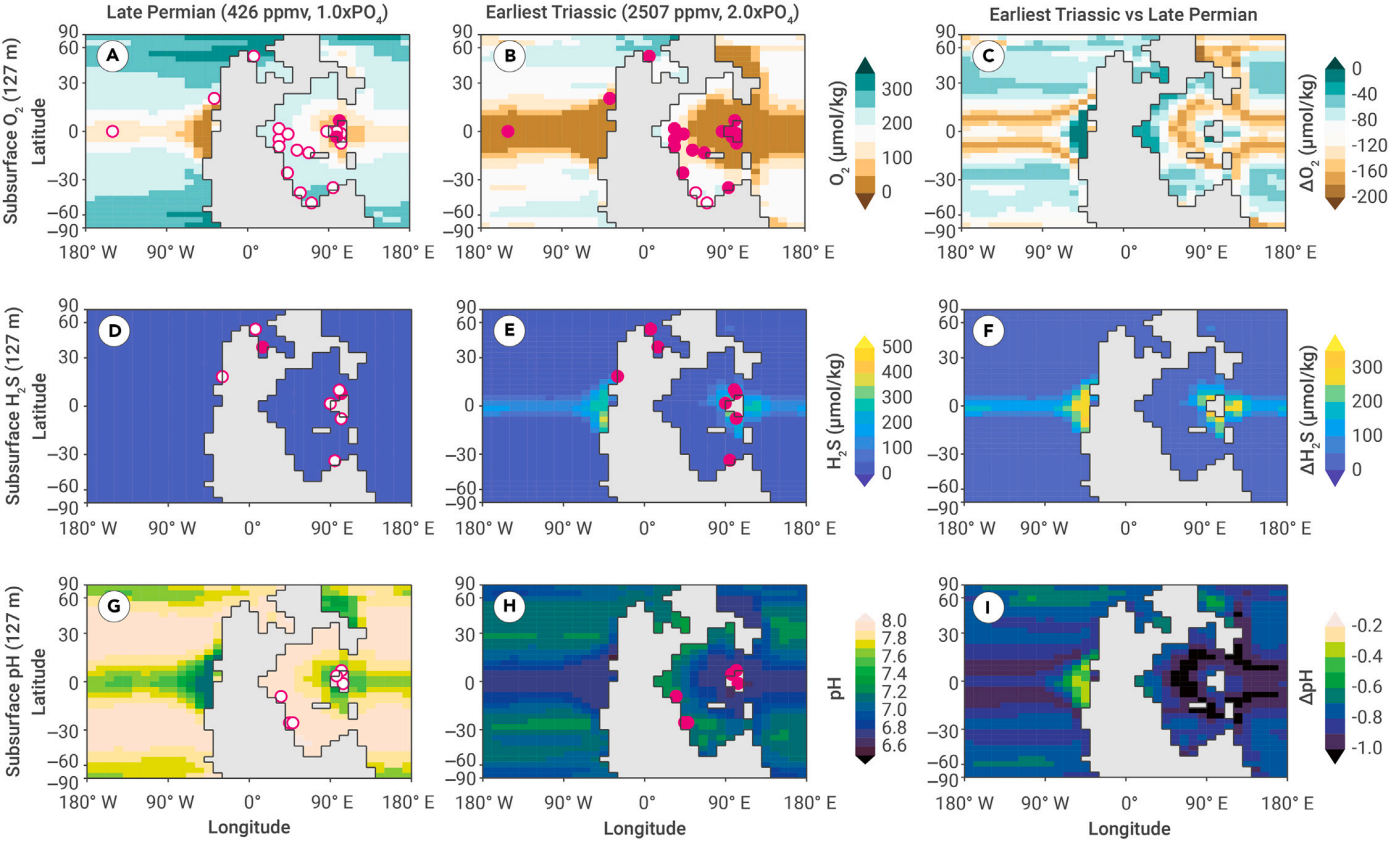
Ocean O_2_, H_2_S, and pH changes during the Permian-Triassic extinction using the cGENIE model and geological records (A and B) Subsurface redox conditions in the late Permian and earliest Triassic. (C) The difference in dissolved O_2_ (ΔO_2_) between the late Permian and earliest Triassic. (D and E) Subsurface hydrogen sulfide concentrations in the late Permian and earliest Triassic. (F) The difference in hydrogen sulfide (ΔH_2_S) between the late Permian and earliest Triassic. (G and H) Subsurface pH values in the late Permian and earliest Triassic. (I) The difference in pH values (ΔpH) between the late Permian and earliest Triassic. We also test other levels of ocean phosphate concentrations in the earliest Triassic (see Figures S5‒S7). Model results for dissolved O_2_, H_2_S, and pH are superimposed by observed proxy data (circles). The solid and hollow circles represent the occurrences of anoxia/hypoxia, euxinia, and acidification or not, respectively (see more information in Table S10).

Selectivity driven by respiratory proteins is interpreted as a consequence of widespread hypoxia that developed during mass extinction and persisted into the earliest Triassic, as indicated by multiple geological proxies and Earth system models (Figures 4A and 4B). Uranium isotopic evidence indicates that the area of seafloor anoxia peaked at 18% in the Induan stage compared to <1% prior to extinction.^29^^,^^30^^,^^31^ New results from cGENIE model simulations (see Tables S5–S7) suggest that dissolved O_2_ in the subsurface seawater (127 m) decreased during this event (Figure 4C). Dissolved O_2_ levels in most of the Tethys and tropical Panthalassa regions were less than 50 μmol/kg (Figure 4B), which would have placed marine ecosystems under stress from O_2_ deficiency. Aerobic habitat loss due to temperature-induced hypoxia was prevalent in the Permian-Triassic extinction interval.^32^^,^^33^^,^^34^^,^^35^ In addition, high temperatures would have caused O_2_ demand to rise for marine animals, even in areas where O_2_ concentration did not decline.^33^^,^^34^ Under both situations, the ability to bind and transport O_2_ is critical to meet the stress of low supply, high demand, or both. The constraints of low O_2_ and high temperatures on metabolism would be strongest for the largest species in each clade, which has been found in the fossil record in the Permian-Triassic extinction interval.^18^

Under hypoxic conditions, animals with hemoglobin and hemocyanin can increase O_2_ transport by enhancing O_2_-binding affinity and/or increasing the globin concentration and hematocrit levels.^23^^,^^36^ Advanced respiratory organs and circulatory systems can help increase the distance and efficiency of O_2_ transportation.^23^^,^^27^ In addition, some mollusks and vertebrates contain myoglobin, which contributes to intracellular O_2_ storage and intercellular facilitated diffusion and thus is an important way for marine animals to cope with short-term severe hypoxia.^22^ Although some metazoans, such as brachiopods and bryozoans, also have respiratory proteins (hemerythrin) in their coelomic fluid, the protein concentration and Hill coefficient of hemerythrin are substantially lower than those of hemoglobin and hemocyanin (Table S1), making it difficult for such animals to survive hypoxic conditions.

Animals that do not have O_2_-carrying proteins can only rely on diffusion to obtain O_2_ from the environment. According to Fick’s law, the diffusion flux is proportional to the concentration gradient and inversely proportional to transport distance. It is difficult for animals to obtain sufficient O_2_ by diffusion in hypoxic habitats, as hypoxia leads to a reduced concentration gradient between seawater and body fluids. These negative effects are greater for larger animals, as the larger the individual is, the more O_2_ is required for growth and metabolism.^37^ Moreover, large individuals have increased transport distance and decreased surface-to-volume ratios, which hinders O_2_ transportation from the water to their cells. This explains why the foraminifera that survived the mass extinction exhibited a significant size reduction (also known as the Lilliput effect) in response to low-O_2_ settings during the Early Triassic.^38^

Hemoglobin and hemocyanin can also compensate for the increased energy budget caused by ocean acidification. During the Permian-Triassic mass extinction, the calcium carbonate saturation state decreased remarkably due to a 6-fold increase in *p*CO_2_,^39^^,^^40^ making it difficult for calcified animals to build shells.^41^^,^^42^ Boron isotope data indicate the acidification of Tethys surface seawater during mass extinction, with an estimated pH drop from ∼8.0 to ∼7.5.^43^ The results from cGENIE support that shallow-seawater (127 m) pH dropped considerably in the Tethys and Panthalassa realms (Figures 4G–4I), decreasing carbonate saturation and having a negative impact on biocalcification.^44^ Under ocean acidification, the energy cost of membrane ATPase to transport H^+^ and Ca^2+^ increases.^45^ An abundant supply of O_2_ and food is indispensable for generating additional ATP to meet the increased energy cost of shell production and metabolism.^46^ Therefore, the presence of hemoglobin or hemocyanin may be the reason why ostracods, mollusks, and vertebrates did not exhibit a significant reduction in body size during mass extinction (Figure 1D). Another advantage of hemoglobin and hemocyanin is their ability to transport CO_2_ via the Bohr effect while enhancing the efficiency of O_2_ transport—a function that hemerythrin does not have.^27^ In addition, in some brachiopods, hemerythrin shows cooperative binding of O_2_ but lacks cooperativity under low pH conditions.^47^

Selectivity of skeletal minerology has also been interpreted as a good predictor of extinction selectivity.^3^^,^^13^^,^^14^ The diversity of organisms without carbonate shells was less affected during mass extinction, as observed in conodonts and fish (Figure 1A). However, among these clades, radiolarians, which have siliceous skeletons and no O_2_-carrying proteins, showed a greater extinction proportion than that of other non-carbonate taxa. In addition, the abundance and diversity of siliceous organisms declined massively, resulting in an Early Triassic “chert gap.”^48^^,^^49^ Hence, O_2_-carrying capacity can explain extinction selectivity when skeletal mineralogy cannot.

The results from the cGENIE model simulations and proxy observations also indicated that sulfidic conditions prevailed in the photic zone, particularly in tropical regions (Figures 4D–4F). Sulfide inhibits respiration and has toxic effects at the whole-organism level.^50^ Sulfide-binding proteins—including hemoglobin—can help marine animals deal with this sulfide build-up. Several proteins play critical roles in sulfide detoxification, including non-enzymatic oxidation by methemoglobin, enzymatic methylation by thiol-S-methyltransferase, and mitochondrial sulfide oxidation by cytochrome *c* oxidase,^50^ thereby contributing to the survival of marine animals in sulfide-rich habitats. Furthermore, the gene expression levels of hemoglobins from the modern bivalve *Lucina pectinata* suggest that sulfide concentrations may participate in the regulation of hemoglobin^51^; this implies that the expression of sulfide-reactive proteins could have been more pronounced in prevailing sulfide environments, such as the Permian-Triassic extinction interval.

This discussion on the role of O_2_-carrying proteins in extinction selectivity is not comprehensive. Some animals express more than one type of O_2_-transport protein.^23^ Here, we use the most dominant types of O_2_-carrying proteins in each clade. Based on the phylogenetic tree of some clades,^52^ we assume that extinct organisms had the same protein type as extant organisms of the same clade. However, we cannot identify the type of O_2_-transport proteins for some extinct clades (such as conodonts and ammonoids) and can only assume that they are of the same protein type as the class/phylum to which they belong. Existing evidence supports this hypothesis; for instance, lagerstätte fossils in Burgess Shale show that extinct trilobites have the same O_2_-carrying protein hemocyanin as extant marine arthropods.^53^ In addition, the body-size and extinction selectivity found in this study support the hypothesis that oceanic anoxia and acidification associated with carbon release and warming from the Siberian Traps and the arc volcanism eruptions in the Tethys and western Panthalassa are the main causes of marine extinctions^54^^,^^55^^,^^56^^,^^57^^,^^58^^,^^59^^,^^60^ but do not exclude the involvement of other environmental events, such as the enhanced weathering and the enrichment of toxic metals (Hg, Cu) found near the Permian-Triassic boundary^4^ (and references therein).

### The role of respiratory protein-driven selectivity in macroevolution and the modern ecological crisis

Our study suggests that protein-based selective extinction was an essential driver of the transition from the Paleozoic to the Modern Evolutionary Fauna. The former consisted mainly of animals with a low O_2_-carrying capacity, including brachiopods, bryozoans, corals, fusulinid foraminifera, and radiolarians,^9^ whose diversity and body size were strongly impacted by the Permian-Triassic mass extinction. In contrast, the Modern Evolutionary Fauna consisted mostly of animals with high O_2_-carrying capacity, including bivalves, gastropods, and fish,^9^ which were less influenced and rebounded rapidly.^10^

The importance of respiratory proteins in understanding extinction selectivity within Paleozoic faunal groups is also shown by the lower impact of the extinction event on the diversity and size of conodonts, cephalopods, and ostracods^9^^,^^61^ (Figure 1), which have hemoglobin or hemocyanin. Furthermore, the strong respiratory protein selectivity of marine animals at the Permian-Triassic transition and their consequent evolutionary paths explain why the ocean acidification and deoxygenation events that occurred during the Mesozoic and Cenozoic^62^ did not affect the general trend of increasing diversity in the Modern Evolutionary Fauna.^9^^,^^63^

Our findings are crucial for providing early warnings of potential ecological crises induced by future global warming and human activities.^64^^,^^65^^,^^66^ As ocean deoxygenation and acidification intensify, the threat of a sixth mass extinction increases,^67^^,^^68^ particularly affecting marine organisms with low O_2_-carrying capacity, including foraminifera, radiolarians, corals, sponges, brachiopods, and bryozoans. These organisms appear to be underrepresented in conservation efforts, with many not being listed on the International Union for Conservation of Nature (IUCN) Red List. Consequently, it is imperative that the IUCN intensifies its monitoring and protection efforts for these vulnerable organisms to avert their potential disappearance in upcoming ecological crises, thus ensuring the preservation of marine ecosystem diversity and balance.

## Materials and methods

See the supplemental information for details.

## Data and code availability

All data are publicly available at Zenodo (https://doi.org/10.5281/zenodo.8079149). Additional data are provided as Figures S1–S7 and Tables S1–S10.

Computer codes for body-size reduction and meta-analysis are publicly available at Zenodo (https://doi.org/10.5281/zenodo.8079149). The code for the cGENIE Earth system model is hosted on GitHub and is available at https://github.com/derpycode/cgenie.muffin. Details of the code installation and basic model configuration can be found in a PDF file (https://www.seao2.info/cgenie/docs/muffin.pdf).

## Lead contact website

https://grzy.cug.edu.cn/songhaijun/en/index.htm

https://www.researchgate.net/profile/Haijun-Song.

## Supplemental information

Document S1. Supplemental materials and methods, Figures S1–S7, and Tables S1–S10

Data S1. Fossil occurrence of marine animals

Data S2. Body size dataset of marine animals in the Changhsingian and Induan stages

Data S3. Predictor variables and extinction status during the Permian-Triassic mass extinction

Data S4. Fossil data used to calculate size reduction for all clades at genus level

Data S5. Fossil data used to calculate size reduction for all clades at species level

Data S6. Fossil data used to calculate size reduction for carbonate clades at genus level

Data S7. Fossil data used to calculate size reduction for carbonate clades at species level

Data S8. Extinction data for meta-analysis of all genera

Data S9. Extinction data for meta-analysis of carbonate

Data S10. Size loss data for meta-analysis of all genera

Data S11. Size loss data for meta-analysis of carbonate

Data S12. Size loss data for meta-analysis of all species

Data S13. Size loss data for meta-analysis of carbonate

Data S14. R code for body size loss

Data S15. R code for meta-analysis

Document S2. Article plus supplemental information
