# Supplementary material for: Respiratory protein-driven selectivity during the Permian-Triassic mass extinction
Source: Innovation (Camb). 2024 Mar 28;5(3):100618. doi: 10.1016/j.xinn.2024.100618 (PMC11025005; doi:10.1016/j.xinn.2024.100618)
Supplement: Document S1. Supplemental materials and methods, Figures S1–S7, and Tables S1–S10 [file mmc1.pdf]

The Innovation, Volume *5*

Supplemental Information

Respiratory protein-driven selectivity during the Permian-Triassic mass extinction

Haijun Song, Yuyang Wu, Xu Dai, Jacopo Dal Corso, Fengyu Wang, Yan Feng, Daoliang Chu, Li Tian, Huyue Song, and William J. Foster

Supplemental Information

Respiratory protein-driven selectivity during the Permian–Triassic mass extinction

Haijun Song*, Yuyang Wu, Xu Dai, Jacopo Dal Corso, Fengyu Wang, Yan Feng, Daoliang Chu, Li Tian, Huyue Song, William J. Foster

Correspondence to: haijunsong@cug.edu.cn

Table of Contents

MATERIALS AND METHODS
- Fossil occurrence data
- Body size data
- Extinction
- Body size reduction
- Logistic regression
- Meta-analysis
- Earth system model simulations

Figs. S1–S7
Tables S1–S10
Captions for Codes S1–S2
Captions for Data S1–S13
References 1–96
Codes S1–S2
Data S1–S13

MATERIALS AND METHODS

Fossil occurrence data

Fossil data used to calculate diversity variation were obtained from a previously published database of Permian–Triassic marine fossils [1,2]. The database contains 52,322 occurrences at the generic level from 1,768 published papers and the Paleobiology Database, spanning the late Permian Changhsingian to the Late Triassic Rhaetian (Data S1). Our analysis is based on the occurrences of genera, as species-level identifications is often inaccurate and inconsistent. For example, a specimen may be species A in Europe and species B in Asia, even though they are the same species. Within the considered interval, a total of 1,097 genera belong to 13 major clades, including two clades of protozoa (foraminifera and radiolarians), nine clades of invertebrates (corals, sponges, brachiopods, bryozoans, ostracods, cephalopods, gastropods, bivalves, and echinoderms), and two clades of vertebrates (conodonts and fishes). For marine arthropods, we used only ostracod data because ostracods are abundant in the fossil record during the late Permian. Other marine arthropods are very rare in this time interval. For example, only two genera of trilobite, one genus of chelicera, and one genus of decapod are recorded in the Changhsingian bin compared to > 100 genera of ostracods in the Paleobiology Database. We did not consider background extinction in the late Permian because many studies have shown that the background extinction rate of marine taxa in the Changhsingian was negligible compared to the mass extinction interval around the Permian–Triassic boundary [3-6]. Therefore, the results using the Changhsingian and Induan fossil data reflect a selectivity pattern of the Permian–Triassic mass extinction rather than background extinction.

Body size data

We updated the comprehensive database of Schaal et al. [7] to assign body size expressed as the maximum length for each species (see Data S2). Using the maximum size for each taxon is a common approach for body size studies, as the effects of

juvenile specimens in the database can be avoided [7-12]. We followed the same methods to compile additional data for taxa not included in this database. A number of recently published databases were used to compile the size data, including references [13-18]. Other size data were mainly obtained from the published taxonomic literature. Only common taxa from both Changhsingian and Induan are included because these taxa have abundant fossil data to study their size change during the Permian-Triassic interval, i.e., foraminifera, brachiopods, ostracods, gastropods, cephalopods, bivalves, conodonts, and fishes. Other taxa, including corals, sponges, radiolarians, bryozoans, and echinoderms, are absent/very rare in the Induan bin and accordingly are not included in this study. The Changhsingian and Induan body size dataset is composed of 1495 species in 635 genera belonging to eight common clades (Data S2).

Extinction

The environmental crisis spanning the Permian–Triassic boundary, e.g., extreme high temperatures [19], ocean anoxia [20-22], and potential ocean acidification [23], was just the start of a series of perturbations that extended throughout the Early Triassic [19,24,25]. Prolonged environmental stress not only exacerbated species extinctions but also negatively affected Early Triassic recovery, which further hampered the rebound of biodiversity [2,26-28]. Here, we focus on the selectivity in extinction of animals with different respiratory proteins.

To investigate selectivity, we used multiple methods to calculate the extinction, i.e., the proportion [29], per capita [29], three timer [30], and gap filler [31] extinction estimators. These estimators can decrease the Signor-Lipps effect and edge effects to some extent [31]. Error bars for the proportion of extinction represent binomial 95% confidence intervals [32]. Considering that the extinction episodes straddled the Permian–Triassic boundary [3-5,33] and lasted approximately 60 kyr from the latest Changhsingian to earliest Induan [34], species that occurred in the extinction interval from the *Clarkina meishanensis* to *Isarcicella staeschei* zones are included in the Changhsingian bin (Figure S2).

Body size reduction

Body size change during the Permian–Triassic mass extinction was analyzed by the Mann–Whitney U test and resampling method. We performed a two-tailed paired Mann–Whitney U test using SPSS (version 25). We used a bootstrap method with 1000 replicates to estimate the size reduction of major clades and oxygen-carrying types. Size reduction between the two adjacent time bins, i.e., Changhsingian and Induan, is represented by the following equation: Size reduction = $\log_{10}(Size_{Ch}) - \log_{10}(Size_{In})$, where $Size_{Ch}$ = median value of Changhsingian body size; $Size_{In}$ = median value of Induan body size. The standard deviation (1 SD) was calculated from 1000 bootstrap replicates of size reduction. Resampling experiments were performed in R. The results of the Mann–Whitney U test and the resampling method matched well (Figure 1). Size data (Data S4–6) were analyzed at both the genus level (Figure 1, Figure S3) and species level (Figure S4). The genus-level analysis of size change parallels the genus-level analysis of mass extinction. The results of the species-level analysis are consistent with the genus-level results, both supporting the significant selectivity among taxa with different oxygen carrying capacities.

Logistic regression

We applied multiple logistic regression models to evaluate the association of physiological and ecological traits with extinction. This analytical approach has been successfully applied to assess extinction selectivity in past and future biodiversity crises [12,35-38]. We performed logistic regression analysis using SPSS (version 25). Logistic regression was performed to evaluate the relationship between physiological and ecological variables and selectivity in extinction. The analysis for evaluating extinction used pre-extinction genera and their extinction (0) and survival (1) as the outcome variables (Data S3).

The logistic regression analysis used mineralogy, oxygen-carrying capacity, physiological buffering capacity, geographic range, motility, species richness and circulatory system as predictors. A multivariate framework for oxygen-carrying capacity, physiological buffering capacity, and motility was used, as it provides a

more detailed approach [39,40](Table S3). Oxygen carrying capacity is a continuous variable, expressed as the maximum O_2-carrying capacity of each oxygen carrier (Table S1). Considering the variation in oxygen carrying capacity for each respiratory protein, we also used the median O_2-carrying capacity to perform a logistic regression analysis. The results showed a consistent selectivity between extinction and O_2-carrying capacity (Tables S4 – S8). There are also uncertainties in diffusion. For example, temperature can affect the rate of diffusion. The physiological buffering capacity category includes no carbonate load and less vulnerable to hypercapnia, moderate carbonate load and some buffering capacity, and heavy carbonate load with little buffering capacity following references [41-45]. The mineralogy category consists of carbonate and non-carbonate following references [36,46]. Silica and phosphate skeletons are not ranked because they are unordered categorical variables and were combined with soft-bodied animals into one variable, i.e., non-carbonate. Motility is composed of fast mobile, slow mobile, facultatively mobile, stationary unattached and stationary attached [40,43]. The circulatory system includes no circulatory system, open circulatory system, and closed circulatory system (see Table S2). The occurrence is the number of occurrences in the fossil database. Geographic range and occurrence are continuous variables and were log-transformed ($\log_e$). The categories of physiological buffering capacity, mineralogy, motility, and circulatory system are ordered factor variables. A correlation matrix of the variables shows that a few variables are correlated, e.g., geographic range and number of occurrences (Table S9). In general, the correlation values are low. Variance inflating factors (VIFs) and tolerances were used to analyze the severity of multicollinearity in the regression analysis (Tables S6, S8). The results of the analysis including all seven variables show a maximum VIF of 10.6 in the physiological buffering capacity category, suggesting significant multicollinearity. This is likely due to the association between variables of the circulatory system and physiological buffering capacity. Therefore, we performed logistic regression after removing the circulatory system. We chose to keep physiological buffering capacity because it is a more common variable for the

analysis of extinction selectivity. The results using six variables with variance VIF < 7 and tolerances > 0.1 (see Table S6) suggest that the estimated coefficient is not inflated by other factors.

Meta-analysis

We used a mixed-effects meta-regression model to perform linear regression between maximum oxygen capacity and proportion of extinction and size reduction [47]. Data that were used to perform the meta-analysis are shown in Data S8 – S11. Different from standard linear regression, this method could consider the sampling variances or uncertainty of data, as our extinction magnitude and size reduction data have uncertainty. This analysis was performed by the function rma in meta for the R package [47]. Regression lines and their 95% confidence intervals, regression coefficients, and *p* values in Figure 1 and Figure S3 are derived from the analysis results of function rma in meta.

Earth system model simulations

The cGENIE, an Earth system model of intermediate complexity, is used to simulate ocean O_2, H_2S, and pH changes during the Permian–Triassic mass extinction. We ran the model on a 36x36 grid with 16 vertical levels in the ocean, with specific modules and boundary conditions taken from previous work [48]. In particular, a temperature-dependent POM remineralization module is applied in this simulation, which describes the temperature-dependent microbial metabolism that has a significant effect on ocean anoxia and euxinia [48,49]. The ocean phosphate concentrate is set as 1.0 and 2.0 times the present oceanic levels (2.159 μmol kg^{-1}) in the late Permian and earliest Triassic, respectively, following reference [48]. We also test other levels of ocean phosphate concentrations in the earliest Triassic (1.0, 1.5, 2.5 and 3.0 × modern levels, see Figs. S5–S7). We only modified the boundary conditions of atmospheric CO_2 levels at the Permian–Triassic boundary, which are different from those set in reference [48]. The pCO_2 in our simulations comes from previous CO_2 reconstruction based on high-resolution $\delta^{13}C$ of C_3 plant remains from terrestrial

sections in southwestern China [50], which is set as 426 ppmv in the late Permian and 2507 ppmv in the earliest Triassic. All experiments are run for 10 kyr to reach the steady state.

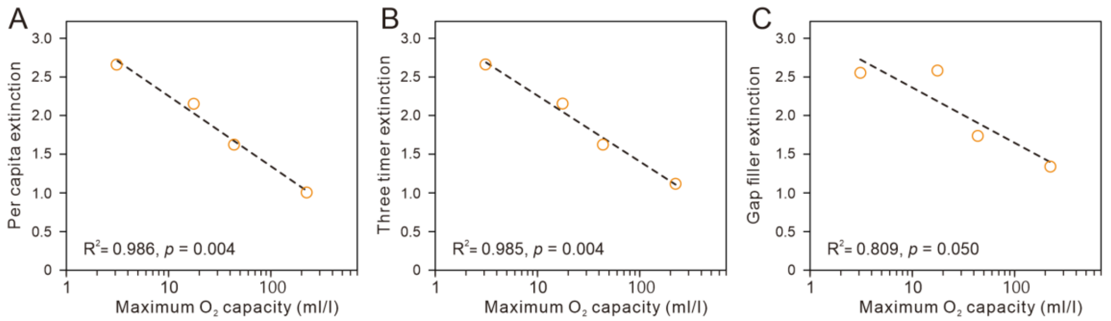

Fig. S1. The relationships between extinction rate and maximum oxygen capacity of marine animals with carbonate shells. (A) Per capita extinction. **(B)** Three timer extinction. **(C)** Gap filler extinction.

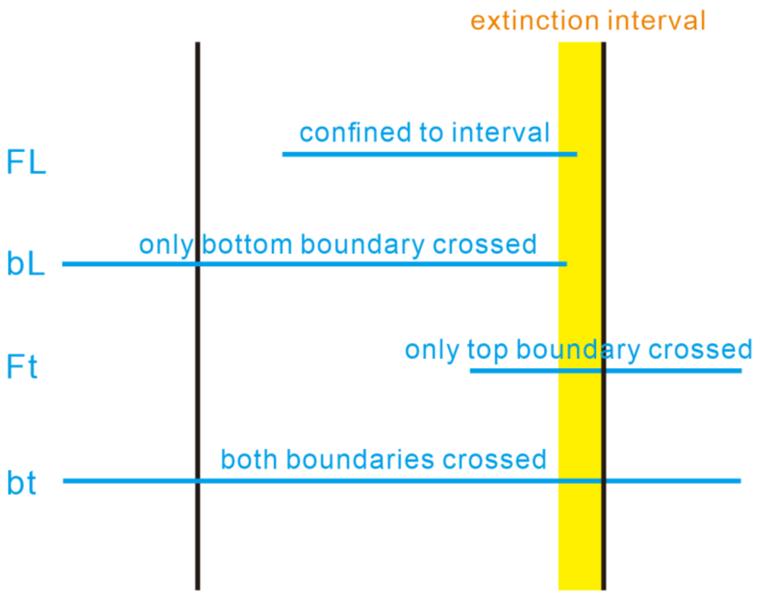

Fig. S2. Four fundamental classes of taxa during stages containing the mass extinction interval. Modified after references [29,51]. The Permian–Triassic extinction interval ranges from the conodont *Clarkina meishanensis* zone to the *Isarcicella staeschei* zone [3,4].

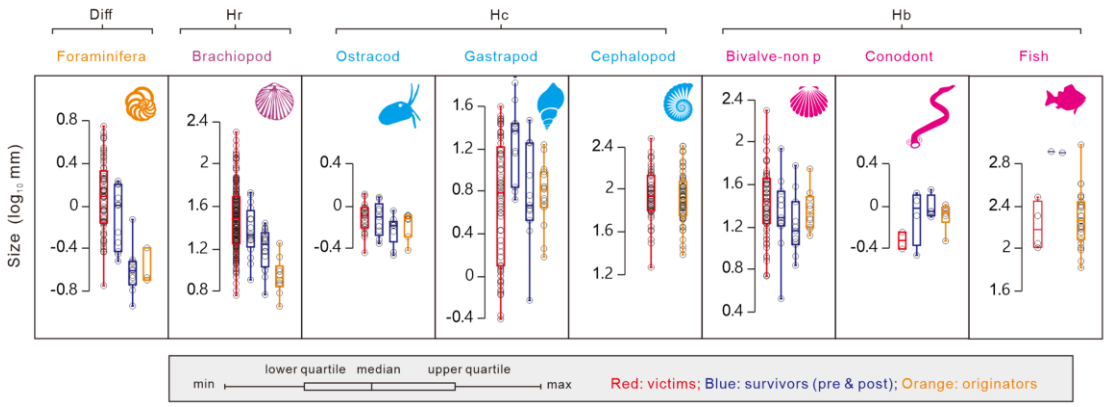

Fig. S3. Changes in body size of marine animals at the genus level during the Permian–Triassic mass extinction. For each clade, four dotplots and boxes represent the size distribution of, from left to right, victims, survivors (pre, Changhsingian), survivors (post, Induan), and new comers (originators). Diff, Hr, Hc, and Hb represent different types of animals that use diffusion, hemoglobin, hemocyanin, and hemoglobin, respectively, to transport oxygen from their surroundings to their bodies. Bivalve-non p represents hemoglobin non-protobranchia bivalve.

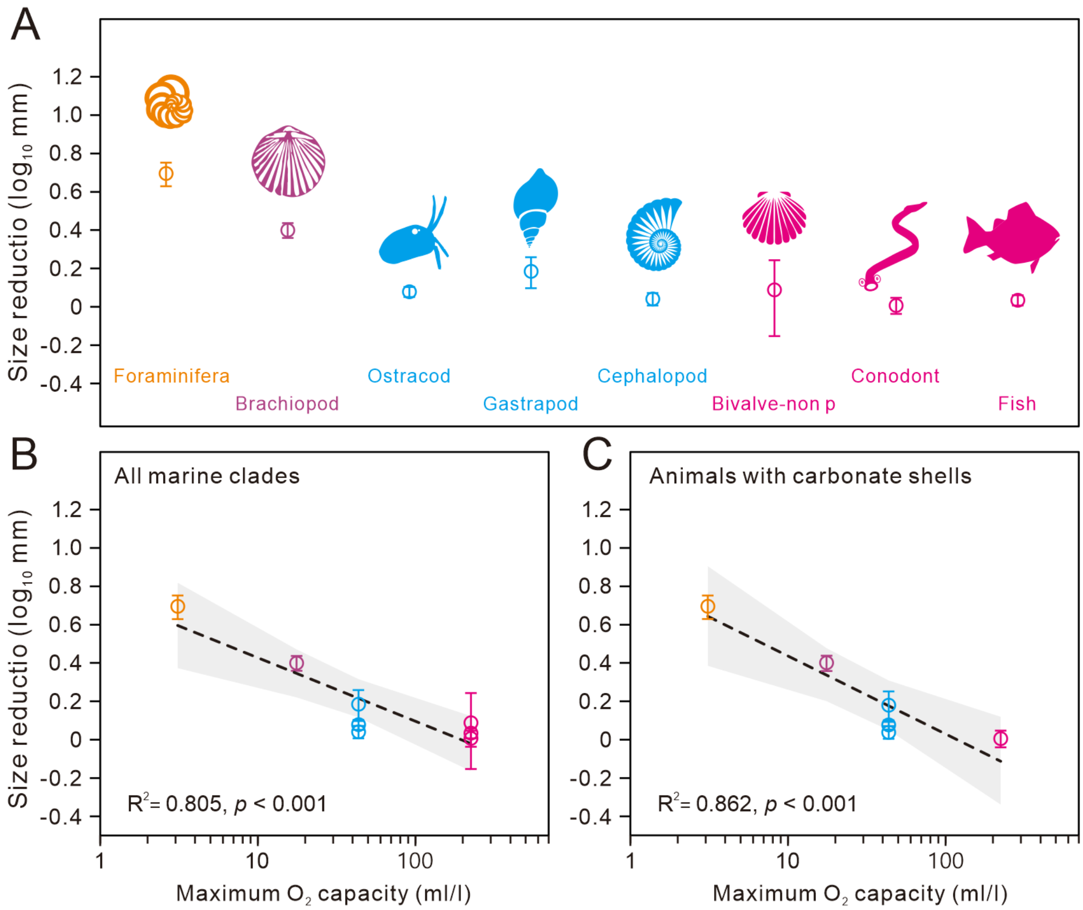

Fig. S4. Changes in the median size of marine animals at the species level during the Permian–Triassic mass extinction. (A) Body size reduction of major marine clades. **(B)** Correlation between size reduction and oxygen capacity for marine clades. **(C)** Correlation between size reduction and oxygen capacity for animals with carbonate shells. Vertical bars represent the standard deviation (1 SD) of 1000 bootstrap replicates. Diff (orange color), Hr (purple color), Hc (blue color), and Hb (magenta color) represent different types of animals that use diffusion, hemerythrin, hemocyanin, and hemoglobin, respectively, to transport oxygen from the surroundings to their bodies. Bivalve-non p represents hemoglobin non-protobranchia bivalve. Dashed lines and shades represent linear regression lines and their 95% confidence intervals. For the methods used to calculate size reduction, see Materials and Methods. For fossil occurrence and size data, see Data 1 and 2.

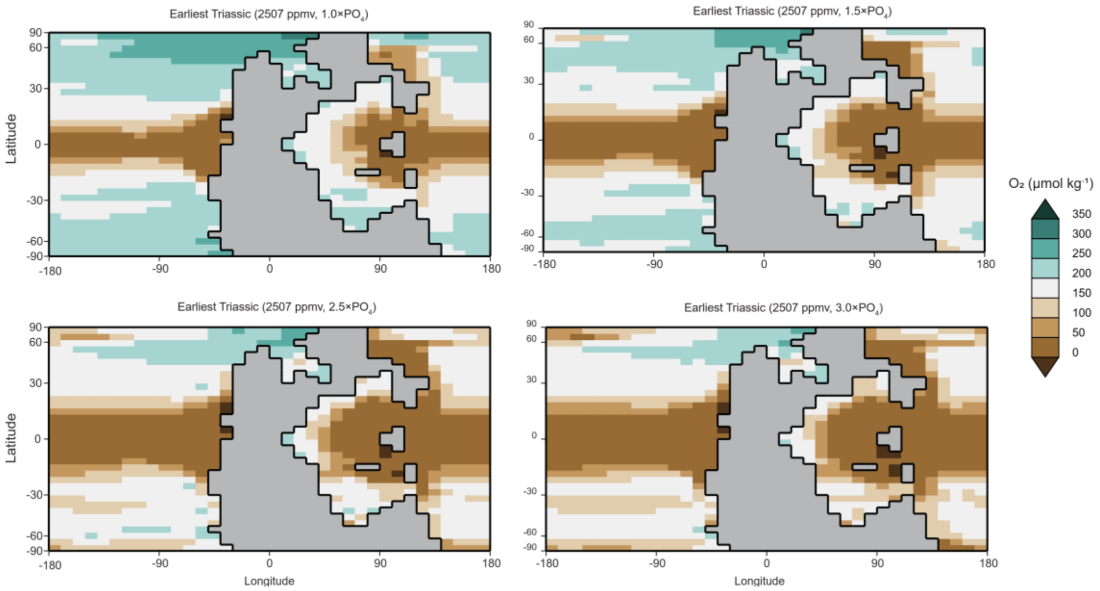

Fig. S5. The cGENIE results of ocean O_2, H_2S, and pH under different levels of phosphate concentration (1.0, 1.5, 2.5 and 3.0 × modern levels) in the earliest Triassic.

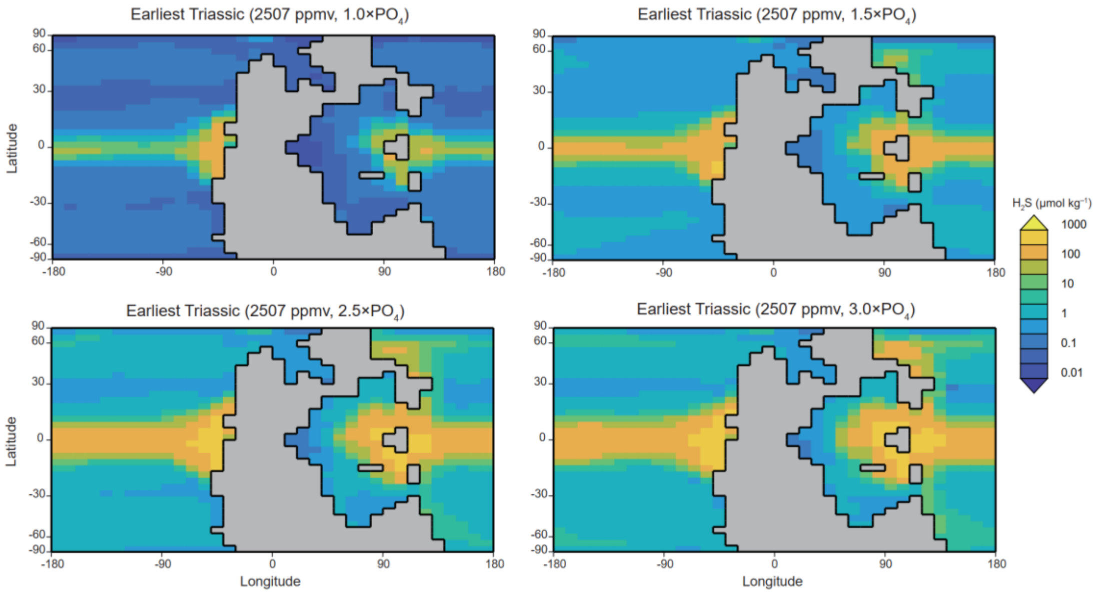

Fig. S6. The cGENIE results of ocean H_2S under different levels of phosphate concentration (1.0, 1.5, 2.5 and 3.0 × modern levels) in the earliest Triassic.

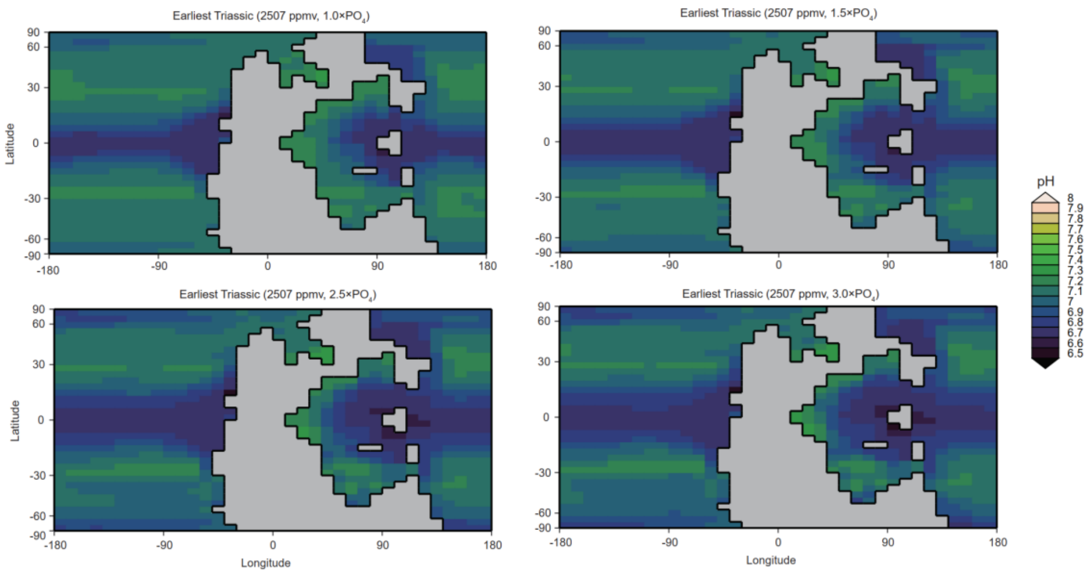

Fig. S7. The cGENIE results of ocean pH under different levels of phosphate concentration (1.0, 1.5, 2.5 and 3.0 × modern levels) in the earliest Triassic.

Table S1. Types and characteristics of oxygen carriers. In this work, we assumed that 250 million-year-old organisms had the same protein type and O_2-carrying capacity as modern organisms of the same clade.

Oxygen carrier	Diffusion	Hemerythrin	Hemocyanin	Hemoglobin	References
Function	O_2 transporting	reversible O_2 binding	reversible O_2 binding	reversible O_2, CO_2 binding	[52]
Animals	protozoa, sponge, coral	brachiopod, bryozoan	protobranchia bivalve, ostracod, gastropod, cephalopod	non-protobranchia bivalve, echinoderm, conodont, fish	Table S2
Metal	/	Fe	Cu	Fe	[52]
Metal : O_2	/	2Fe : O_2	2Cu : O_2	Fe : O_2	[52]
Color	/	burgundy	blue	red	[52]
Position	/	plasma	hemolymph	red blood cell	[52]
Molecular mass	/	108 kDa	400-9000 kDa	64 KDA	[52]
Molecular mass of subunit/functional unit	/	13.5 kDa	50-72 kDa	16 kDa	[53,54]
Protein concentration	/	~10.6 g/L	20-140 g/L	120-160 g/L	[53-55]
Oxygen capacity	3.1 mL/L	~17.6 mL/L	9.0-43.6 mL/L	168-224 mL/L	[53,56,57]
Hill coefficient	/	1-1.78	1.6-3.0	2.8-3.0	[58-60]
Number of subunits	/	8	many	4	[61]

Table S2. Types of oxygen carriers and circulatory systems in animals. (A) Types of oxygen carriers. **(B)** Types of circulatory systems.

A	Clades	Oxygen carriers	References
	foraminifera	diffusion	62,63
	radiolarian	diffusion	63
	sponge	diffusion	64
	coral	diffusion	65
	brachiopod	hemerythrin	66-70
	bryozoan	hemerythrin	67,71
	gastropod	hemocyanin	72-74
	ostracod	hemocyanin	75
	cephalopod	hemocyanin	76-78
	protobranchia bivalve	hemocyanin	79-81
	non-protobranchia bivalve	hemoglobin	82-84
	fish	hemoglobin	85-87
	echinoderm-Holothuroidea	hemoglobin	88,89
	echinoderm-Echinoidea	hemoglobin	90
	echinoderm-Ophiuroidea	hemoglobin	91-93

B	Clades	Circulatory systems	References
	foraminifera	non	94,95
	radiolarian	non	94,95
	sponge	non	94-96
	coral	non	94-96
	brachiopod	open	94,95,97
	gastropod	open	94-97
	ostracod	open	94-97
	bivalve	open	94-97
	cephalopod	closed	94-97
	fish	closed	94-97

Table S3. Ecological traits used to analyze the selectivity of extinction. Physiological buffering capacity after references [41-45]. Shell mineralogy after references [36,46]. Silica and phosphate skeletons are not ranked because they are unordered categorical variables, and were combined into one variable, i.e., non-carbonate. Motility after references [40,43]. The values of O_2-carrying capacity and circulatory system types are from references in Tables S1 and S2. Geographic range and abundance are continuous variables applied in natural logarithmic form.

**Physiological buffering capacity**
1 Heavy carbonate load & no buffering capacity
2 Moderate carbonate load & moderate buffering capacity
3 Little or no carbonate load & strong buffering capacity
**Shell mineralogy**
0 Carbonate
1 Silica & phosphate
**Motility**
1 Stationary & attached
2 Stationary & unattached
3 Facultatively motile
4 Motile & slow
5 Motile & fast
**Circulatory system**
0 No circulatory system
1 Open circulatory system
2 Closed circulatory system
**Oxygen carrying capacity**
Diffusion: logarithmic (maximum O_2 capacity) = 0.49 $\log_{10}$ ml/l
Hemerythrin: logarithmic (maximum O_2 capacity) = 1.25 $\log_{10}$ ml/l
Hemocyanin: logarithmic (maximum O_2 capacity) = 1.64 $\log_{10}$ ml/l
Hemoglobin: logarithmic (maximum O_2 capacity) = 2.35 $\log_{10}$ ml/l
**Geographic range**
Natural logarithmic (number of equal-area geographic grid-cells)
**Occurrence**
Natural logarithmic (number of occurrences)

Table S4. Results of logistic regression for extinction. (A) Results of logistic regression including all six variables. **(B)** Results of logistic regression including four variables that have significant logistic regression relationships in panel A. **(C)** Results of logistic regression with O_2-carrying capacity as a categorical covariate. OCC, maximum value of oxygen carrying capacity; SE, standard error; Exp, exponential; CI, confidence interval.

**A**	Variable	Coefficient	SE	Wald χ^2	*p* value	Exp (coefficient)	Lower 95% CI	Upper 95% CI
	Minerology	0.680	0.574	1.406	0.236	1.975	0.641	6.079
	OCC	1.418	0.220	41.429	0.000	4.129	2.681	6.359
	Motility	-0.209	0.079	6.952	0.008	0.812	0.695	0.948
	Physiological buffering capacity	0.914	0.343	7.100	0.008	2.494	1.273	4.884
	Geographic range	1.244	0.371	11.269	0.001	3.471	1.678	7.177
	Number of occurrence	0.058	0.231	0.063	0.802	1.060	0.674	1.665

**B**	Variable	Coefficient	SE	Wald χ^2	*p* value	Exp (coefficient)	Lower 95% CI	Upper 95% CI
	OCC	1.297	0.188	47.377	0.000	3.657	2.528	5.290
	Motility	-0.237	0.075	9.982	0.002	0.789	0.681	0.914
	Physiological buffering capacity	1.275	0.163	61.433	0.000	3.579	2.602	4.923
	Geographic range	1.324	0.157	70.741	0.000	3.760	2.762	5.120

**C**	Variable	Coefficient	SE	Wald χ^2	*p* value	Exp (coefficient)	Lower 95% CI	Upper 95% CI
	Hemerythrin	1.373	0.420	10.702	0.001	3.946	1.734	8.983
OCC	Hemocyanin	1.263	0.468	7.286	0.007	3.536	1.413	8.846
	Hemoglobin	2.362	0.367	41.504	0.000	10.615	5.174	21.780
	Motility	-0.162	0.112	2.086	0.149	0.850	0.682	1.060
	Physiological buffering capacity	1.362	0.203	45.013	0.000	3.905	2.623	5.814
	Geographic range	1.330	0.159	69.704	0.000	3.780	2.767	5.165

Table S5. Results of logistic regression for extinction using median values of oxygen carrying capacity. OCC, median value of oxygen carrying capacity; SE, standard error; Exp, exponential; CI, confidence interval.

Variable	Coefficient	SE	Wald χ^2	*p* value	Exp (coefficient)	Lower 95% CI	Upper 95% CI
Minerology	0.319	0.548	0.339	0.560	1.376	0.470	4.033
OCC	1.353	0.210	41.335	0.000	3.867	2.561	5.841
Motility	-0.161	0.078	4.301	0.038	0.851	0.731	0.991
Physiological buffering capacity	1.085	0.334	10.577	0.001	2.961	1.539	5.694
Geographic range	1.219	0.369	10.903	0.001	3.385	1.641	6.981
Number of occurrence	0.069	0.230	0.090	0.765	1.071	0.682	1.683

Table S6. Results of multicollinearity diagnostics. OCC, oxygen carrying capacity; SE, standard error; VIF, variance inflating factor.

Model	Unstandardized coefficients B	Unstandardized Coefficients SE	Standardized coefficients Beta	t	*p* value	Tolerance	VIF
(Constant)	-0.203	0.040		-5.051	0.000		
Minerology	0.208	0.072	0.196	2.871	0.004	0.196	5.104
OCC	0.164	0.024	0.290	6.843	0.000	0.509	1.963
Motility	-0.019	0.010	-0.080	-1.879	0.061	0.501	1.996
Physiological buffering capacity	0.041	0.039	0.081	1.056	0.291	0.157	6.364
Geographic range	0.109	0.033	0.232	3.299	0.001	0.185	5.405
Number of occurrence	0.017	0.021	0.054	0.773	0.440	0.188	5.333

Table S7. Results of logistic regression including all seven variables. OCC, oxygen carrying capacity; SE, standard error; Exp, exponential; CI, confidence interval.

Variable	Coefficient	SE	Wald χ^2	*p* value	Exp (coefficient)	Lower 95% CI	Upper 95% CI
OCC	1.797	0.387	21.528	0.000	6.033	2.824	12.889
Minerology	0.579	0.810	0.512	0.474	1.785	0.365	8.726
Geographic range	0.962	0.380	6.417	0.011	2.616	1.243	5.507
Physiological buffering capacity	0.808	0.446	3.285	0.070	2.244	0.936	5.379
Motility	-0.053	0.122	0.188	0.664	0.948	0.746	1.205
Number of occurrence	0.239	0.240	0.993	0.319	1.270	0.794	2.030
Circulatory system	-0.971	0.405	5.733	0.017	0.379	0.171	0.839

Table S8. Results of multicollinearity diagnostics including all seven variables. OCC, oxygen carrying capacity; SE, standard error; VIF, variance inflating factor.

Model	Unstandardized coefficients B	Unstandardized Coefficients SE	Standardized coefficients Beta	t	*p* value	Tolerance	VIF
(Constant)	-0.224	0.040		-5.542	0.000		
OCC	0.206	0.044	0.373	4.723	0.000	0.161	6.226
Minerology	0.077	0.102	0.071	0.752	0.453	0.111	9.018
Geographic range	0.083	0.034	0.183	2.471	0.014	0.182	5.481
Physiological buffering capacity	0.077	0.052	0.151	1.468	0.143	0.095	10.576
Motility	-0.013	0.014	-0.057	-0.936	0.349	0.271	3.696
Number of occurrence	0.029	0.022	0.099	1.342	0.180	0.185	5.396
Circulatory system	-0.120	0.036	-0.225	-3.336	0.001	0.220	4.547

Table S9. Correlation matrix of the investigated variables during the extinction. OCC, oxygen carrying capacity.

	Extinction	OCC	Minerology	Geographic range	Physiological buffering capacity	Motility	Number of occurrence	Circulatory system
Extinction	1							
OCC	0.213	1						
Minerology	0.253	0.087	1					
Geographic range	0.259	-0.135	0.051	1				
Physiological buffering capacity	0.299	0.469	0.778	-0.010	1			
Motility	0.074	0.572	0.121	-0.041	0.516	1		
Number of occurrence	0.250	-0.126	0.015	0.896	-0.036	-0.067	1	
Circulatory system	0.015	0.790	-0.058	-0.114	0.338	0.717	-0.090	1

Table S10. Observed proxy data in the late Permian and Permian–Triassic mass extinction (PTME).

Locality	Phase	Anoxia/hypoxia	Euxinia	Acidification	References
South China (Meishan)	Late Permian	Yes	Yes	–	98-100
South China (Meishan)	PTME	Yes	Yes	–	98-105
South China (Huangzhishan)	Late Permian	No	No	–	103
South China (Huangzhishan)	PTME	Yes	Yes	–	103
South China (Dawen)	Late Permian	No	–	–	106
South China (Dawen)	PTME	Yes	–	–	106
South China (Dajiang)	Late Permian	No	–	–	107,108
South China (Dajiang)	PTME	Yes	–	–	107,108
South China (Laolongdong)	Late Permian	No	–	–	109
South China (Laolongdong)	PTME	Yes	–	–	109
South China (Cili)	Late Permian	No	–	–	110
South China (Cili)	PTME	Yes	–	–	110
South China (Taiping)	Late Permian	Yes	–	–	111
South China (Taiping)	PTME	Yes	–	–	111
South China (Sichuan)	Late Permian	–	No	–	112
South China (Sichuan)	PTME	–	Yes	–	112
South China (Chaohu)	Late Permian	No	No	–	103
South China (Chaohu)	PTME	Yes	Yes	–	103
South China (Meishan)	Late Permian	–	–	No	113
South China (Meishan)	PTME	–	–	Yes	113
South China (Dajiang)	Late Permian	–	–	No	114
South China (Dajiang)	PTME	–	–	Yes	114
South China (Jianzhishan)	Late Permian	–	–	No	115
South China (Jianzhishan)	PTME	–	–	Yes	115
Italy	Late Permian	–	–	No	23,116
Italy	PTME	–	–	Yes	23,116
Oman	Late Permian	–	–	No	116
Oman	PTME	–	–	Yes	116
United Arab Emirates	Late Permian	–	–	No	117
United Arab Emirates	PTME	–	–	Yes	117
Vietnam	Late Permian	No	No		118,119
Vietnam	PTME	Yes	Yes		118,119
Japan (Kamura)	Late Permian	No	–	–	120
Japan (Kamura)	PTME	Yes	–	–	120
Italy	Late Permian	No	–	–	20,121,122
Italy	PTME	Yes	–	–	20,121,122
Austria	Late Permian	No	–		123
Austria	PTME	Yes	–		123
Hungary	Late Permian	No			124
Hungary	PTME	Yes			124
Arabian Margin	Late Permian	No			125
Arabian Margin	PTME	Yes			125
Slovenia	Late Permian	No			126
Slovenia	PTME	Yes			126
Turkey (Taşkent)	Late Permian	No	–	–	107
Turkey (Taşkent)	PTME	Yes	–	–	107
Iran (Zal)	Late Permian	No	–	–	25
Iran (Zal)	PTME	Yes	–	–	25
Salt Range	Late Permian	No	–	–	127,128
Salt Range	PTME	No	–	–	127,128
South Tibet	Late Permian	No	–	–	129
South Tibet	PTME	No	–	–	129
Western Australia (Perth Basin)	Late Permian	No	No	–	98,124
Western Australia (Perth Basin)	PTME	Yes	Yes	–	98,124
Svalbard	Late Permian	No	No		124,130
Svalbard	PTME	Yes	Yes	–	124,130
Greenland	Late Permian	–	Yes	–	131
Greenland	PTME	–	Yes	–	131
British Columbia	Late Permian	No	–	–	127,129
British Columbia	PTME	Yes	–	–	127,129
Canada (Peace River Basin)	Late Permian	–	No	–	132
Canada (Peace River Basin)	PTME	–	Yes	–	132

Other Supplementary Materials for this manuscript include the following:

Code S1. R code for body size loss.

Code S2. R code for meta-analysis.

Data S1. Fossil occurrence of marine animals.

Data S2. Body size dataset of marine animals in the Changhsingian and Induan stages.

Data S3. Predictor variables and extinction status during the Permian–Triassic mass extinction.

Data S4. Fossil data used to calculate size reduction for all clades at genus level.

Data S5. Fossil data used to calculate size reduction for all clades at species level.

Data S6. Fossil data used to calculate size reduction for carbonate clades at genus level.

Data S7. Fossil data used to calculate size reduction for carbonate clades at species level.

Data S8. Extinction data for meta-analysis all.

Data S9. Extinction data for meta-analysis carbonate.

Data S10. Size loss data for meta-analysis SLdata_genus.

Data S11. Size loss data for meta-analysis SLdata carbonate_genus.

Data S12. Size loss data for meta-analysis SLdata_species.

Data S13. Size loss data for meta-analysis SLdata carbonate_species.

See separate files.

References

1. Song, H., Huang, S., Jia, E., et al. (2020). Flat latitudinal diversity gradient caused by the Permian–Triassic mass extinction. Proc. Natl. Acad. Sci. USA **117**(30):17578—17583. DOI: 10.1073/pnas.1918953117.
2. Song, H., Wignall, P.B., and Dunhill, A.M. (2018). Decoupled taxonomic and ecological recoveries from the Permo-Triassic extinction. Sci. Adv. **4**(10):eaat5091. DOI: 10.1126/sciadv.aat5091.
3. Shen, S., Crowley, J.L., Wang, Y., et al. (2011). Calibrating the end-Permian mass extinction. Science **334**(6061):1367—1372. DOI: 10.1126/science.1213454.
4. Song, H., Wignall, P.B., Tong, J., and Yin, H. (2013). Two pulses of extinction during the Permian-Triassic crisis. Nat. Geosci. **6**(1):52—56. DOI: 10.1038/ngeo1649.
5. Yin, H., Feng, Q., Lai, X., et al. (2007). The protracted Permo-Triassic crisis and multi-episode extinction around the Permian-Triassic boundary. Global Planet. Change **55**(1—3):1—20. DOI: 10.1016/j.gloplacha.2006.06.005.
6. Fan, J.-x., Shen, S.-z., Erwin, D.H., et al. (2020). A high-resolution summary of Cambrian to Early Triassic marine invertebrate biodiversity. Science **367**(6475):272—277. DOI: 10.1126/science.aax4953.
7. Schaal, E.K., Clapham, M.E., Rego, B.L., et al. (2016). Comparative size evolution of marine clades from the Late Permian through Middle Triassic. Paleobiology **42**(1):127—142. DOI: 10.1017/pab.2015.36.
8. Stanley, S.M. (1973). An explanation for Cope's rule. Evolution **27**(1):1—26. DOI: 10.2307/2407115.
9. Jablonski, D. (1997). Body-size evolution in Cretaceous molluscs and the status of Cope's rule. Nature **385**(6613):250—252. DOI: 10.1038/385250a0.
10. Lockwood, R. (2005). Body size, extinction events, and the early Cenozoic record of veneroid bivalves: a new role for recoveries? Paleobiology **31**(4):578—590. DOI: 10.1666/0094-8373(2005)031[0578:BSEEAT]2.0.CO;2.
11. Heim, N.A., Knope, M.L., Schaal, E.K., et al. (2015). Cope's rule in the evolution of marine animals. Science **347**(6224):867—870. DOI: 10.1126/science.1260065.

12. Payne, J.L., Bush, A.M., Heim, N.A., et al. (2016). Ecological selectivity of the emerging mass extinction in the oceans. Science **353**(6305):1284—1286. DOI: 10.1126/science.aaf2416.

13. Romano, C., Koot, M.B., Kogan, I., et al. (2016). Permian–Triassic Osteichthyes (bony fishes): diversity dynamics and body size evolution. Biol. Rev. **91**(1):106—147. DOI: 10.1111/brv.12161.

14. Shi, G.R., Zhang, Y.-c., Shen, S.-z., and He, W.-h. (2016). Nearshore–offshore–basin species diversity and body size variation patterns in Late Permian (Changhsingian) brachiopods. Palaeogeogr., Palaeoclimatol., Palaeoecol. **448**:96—107. DOI: 10.1016/j.palaeo.2015.07.046.

15. Chen, J., Song, H., He, W., et al. (2019). Size variation of brachiopods from the Late Permian through the Middle Triassic in South China: Evidence for the Lilliput Effect following the Permian-Triassic extinction. Palaeogeogr., Palaeoclimatol., Palaeoecol. **519**:248—257. DOI: 10.1016/j.palaeo.2018.07.013.

16. Feng, Y., Song, H., and Bond, D.P.G. (2020). Size variations in foraminifers from the early Permian to the Late Triassic: implications for the Guadalupian–Lopingian and the Permian–Triassic mass extinctions. Paleobiology **46**(4):511—532. DOI: 10.1017/pab.2020.37.

17. Foster, W., Gliwa, J., Lembke, C., et al. (2020). Evolutionary and ecophenotypic controls on bivalve body size distributions following the end-Permian mass extinction. Global Planet. Change **185**:103088. DOI: 10.1016/j.gloplacha.2019.103088.

18. Foster, W., Lehrmann, D., Yu, M., et al. (2018). Persistent environmental stress delayed the recovery of marine communities in the aftermath of the latest Permian mass extinction. Paleoceanogr. Paleoclimatol. **33**(4):338—353. DOI: 10.1002/2018PA003328.

19. Sun, Y., Joachimski, M.M., Wignall, P.B., et al. (2012). Lethally hot temperatures during the Early Triassic greenhouse. Science **338**(6105):366—370. DOI: 10.1126/science.1224126.

20. Wignall, P.B., and Twitchett, R.J. (1996). Oceanic anoxia and the end-Permian mass extinction. Science **272**(5265):1155—1158. DOI: 10.1126/science.272.5265.1155.

21. Isozaki, Y. (1997). Permo-Triassic boundary superanoxia and stratified superocean: records from lost deep sea. Science **276**:235—238. DOI: 10.1126/science.276.5310.235.

22. Penn, J.L., Deutsch, C., Payne, J.L., and Sperling, E.A. (2018). Temperature-dependent hypoxia explains biogeography and severity of end-Permian marine mass extinction. Science **362**(6419):eaat1327. DOI: 10.1126/science.aat1327.

23. Jurikova, H., Gutjahr, M., Wallmann, K., et al. (2020). Permian–Triassic mass extinction pulses

driven by major marine carbon cycle perturbations. Nat. Geosci. **13**(11):745─750. DOI: 10.1038/s41561-020-00646-4.

24. Payne, J.L., Lehrmann, D.J., Wei, J., et al. (2004). Large perturbations of the carbon cycle during recovery from the end-Permian extinction. Science **305**(5683):506─509. DOI: 10.1126/science.1097023.

25. Zhang, F., Romaniello, S.J., Algeo, T.J., et al. (2018). Multiple episodes of extensive marine anoxia linked to global warming and continental weathering following the latest Permian mass extinction. Sci. Adv. **4**(4):e1602921. DOI: 10.1126/sciadv.1602921.

26. Stanley, S.M. (2009). Evidence from ammonoids and conodonts for multiple Early Triassic mass extinctions. Proc. Natl. Acad. Sci. USA **106**(36):15264─15267. DOI: 10.1073/pnas.0907992106.

27. Foster, W.J., Danise, S., Price, G.D., and Twitchett, R.J. (2017). Subsequent biotic crises delayed marine recovery following the late Permian mass extinction event in northern Italy. PLoS ONE **12**(3):e0172321. DOI: 10.1371/journal.pone.0172321.

28. Luo, M., Buatois, L., Shi, G.R., and Chen, Z.-Q. (2021). Infaunal response during the end-Permian mass extinction. GSA Bulletin **133**(1-2):91-99. DOI: 10.1130/B35524.1.

29. Foote, M. (2000). Origination and extinction components of taxonomic diversity: Paleozoic and post-Paleozoic dynamics. Paleobiology **26**(4):578─605. DOI: 10.1666/0094-8373(2000)026<0578:OAECOT>2.0.CO;2.

30. Alroy, J. (2008). Dynamics of origination and extinction in the marine fossil record. Proc. Natl. Acad. Sci. USA **105**(Supplement 1):11536─11542. DOI: 10.1073/pnas.0802597105.

31. Alroy, J. (2014). Accurate and precise estimates of origination and extinction rates. Paleobiology **40**(3):374─397. DOI: 10.1666/13036.

32. Raup, D. (1991). The future of analytical paleobiology (Paleontological Society, Knoxville, Tennessee).

33. Wang, Y., Sadler, P.M., Shen, S.-z., et al. (2014). Quantifying the process and abruptness of the end-Permian mass extinction. Paleobiology **40**(1):113─129. DOI: 10.1666/13022.

34. Burgess, S.D., Bowring, S., and Shen, S.-z. (2014). High-precision timeline for Earth’s most severe extinction. Proc. Natl. Acad. Sci. USA **111**(9):3316─3321. DOI: 10.1073/pnas.1317692111.

35. Payne, J.L., and Finnegan, S. (2007). The effect of geographic range on extinction risk during

background and mass extinction. Proc. Natl. Acad. Sci. USA **104**(25):10506–10511. DOI: 10.1073/pnas.0701257104.

36. Clapham, M.E., and Payne, J.L. (2011). Acidification, anoxia, and extinction: A multiple logistic regression analysis of extinction selectivity during the Middle and Late Permian. Geology **39**(11):1059–1062. DOI: 10.1130/g32230.1.

37. Finnegan, S., Payne, J.L., and Wang, S.C. (2008). The Red Queen revisited: reevaluating the age selectivity of Phanerozoic marine genus extinctions. Paleobiology **34**(3):318–341. DOI: 10.1666/07008.1.

38. Finnegan, S., Heim, N.A., Peters, S.E., and Fischer, W.W. (2012). Climate change and the selective signature of the Late Ordovician mass extinction. Proc. Natl. Acad. Sci. USA **109**(18):6829–6834. DOI: 10.1073/pnas.1117039109.

39. Dunhill, A.M., Foster, W.J., Sciberras, J., and Twitchett, R.J. (2018). Impact of the Late Triassic mass extinction on functional diversity and composition of marine ecosystems. Palaeontology **61**(1):133–148. DOI: 10.1111/pala.12332.

40. Foster, W.J., Ayzel, G., Münchmeyer, J., et al. (2022). Machine learning identifies ecological selectivity patterns across the end-Permian mass extinction. Paleobiology **48**(3):357–371. DOI: 10.1017/pab.2022.1.

41. Knoll, A.H., Bambach, R.K., Payne, J.L., et al. (2007). Paleophysiology and end-Permian mass extinction. Earth Planet. Sci. Lett. **256**(3–4):295–313. DOI: 10.1016/j.epsl.2007.02.018.

42. Knoll, A.H., Bambach, R.K., Canfield, D.E., and Grotzinger, J.P. (1996). Comparative Earth History and Late Permian Mass Extinction. Science **273**(5274):452–457. DOI: 10.1126/science.273.5274.452.

43. Bambach, R.K., Knoll, A.H., and Sepkoski, J.J., Jr. (2002). Anatomical and ecological constraints on Phanerozoic animal diversity in the marine realm. Proc. Natl. Acad. Sci. USA **99**(10):6854–6859. DOI: 10.1073/pnas.092150999.

44. Kiessling, W., and Simpson, C. (2011). On the potential for ocean acidification to be a general cause of ancient reef crises. Global Change Biol. **17**(1):56–67. DOI: 10.1111/j.1365-2486.2010.02204.x.

45. Payne, J.L., Bush, A.M., Chang, E.T., et al. (2016). Extinction intensity, selectivity and their

combined macroevolutionary influence in the fossil record. Biol. Lett. **12**(10):20160202. DOI: 10.1098/rsbl.2016.0202.

46. Dal Corso, J., Song, H., Callegaro, S., et al. (2022). Environmental crises at the Permian–Triassic mass extinction. Nat. Rev. Earth Eviron. **3**(3):197─214. DOI: 10.1038/s43017-021-00259-4.

47. Viechtbauer, W. (2010). Conducting meta-analyses in R with the metafor package. J. Stat. Softw. **36**(3):1─48. DOI: 10.18637/jss.v036.i03.

48. Hülse, D., Lau, K.V., van de Velde, S.J., et al. (2021). End-Permian marine extinction due to temperature-driven nutrient recycling and euxinia. Nat. Geosci. **14**(11):862─867. DOI: 10.1038/s41561-021-00829-7.

49. Crichton, K.A., Wilson, J.D., Ridgwell, A., and Pearson, P.N. (2021). Calibration of temperature-dependent ocean microbial processes in the cGENIE.muffin (v0.9.13) Earth system model. Geosci. Model Dev. **14**(1):125─149. DOI: 10.5194/gmd-14-125-2021.

50. Wu, Y., Chu, D., Tong, J., et al. (2021). Six-fold increase of atmospheric pCO_2 during the Permian–Triassic mass extinction. Nat. Commun. **12**(1):2137. DOI: 10.1038/s41467-021-22298-7.

51. Foote, M., Miller, A.I., Raup, D.M., and Stanley, S.M. (2007). Principles of Paleontology (Macmillan).

52. Klotz, I.M., and Kurtz Jr, D.M. (1984). Binuclear oxygen carriers: hemerythrin. Acc. Chem. Res. **17**(1):16─22. DOI: 10.1021/ar00097a003.

53. Klotz, I.M., and Keresztes-Nagy, S. (1963). Hemerythrin: molecular weight and dissociation into subunits. Biochemistry **2**(3):445─452. DOI: 10.1021/bi00903a008.

54. Coates, C.J., and Decker, H. (2017). Immunological properties of oxygen-transport proteins: hemoglobin, hemocyanin and hemerythrin. Cell. Mol. Life Sci. **74**(2):293─317. DOI: 10.1007/s00018-016-2326-7.

55. Kamerling, J.P., and Vliegenthart, J.F. (1997). Hemocyanins. In New Comprehensive Biochemistry, (Elsevier), pp. 123─142.

56. Sanders, N., and Childress, J. (1990). Adaptations to the deep-sea oxygen minimum layer: oxygen binding by the hemocyanin of the bathypelagic mysid, Gnathophausia ingens Dohrn. Biol. Bull. **178**(3):286─294. DOI: 10.2307/1541830.

57. Grippi, M.A. (2020). How to calculate the total oxygen content of arterial blood.

58. Lamy, J., Lamy, J., Bonaventura, J., and Bonaventura, C. (1980). Structure, function, and assembly in the hemocyanin system of the scorpion *Androctonus australis*. Biochemistry **19**(13):3033–3039. DOI: 10.1021/bi00554a031.
59. Makino, N. (1989). Hemocyanin from *Tachypleus gigas*. II. Cooperative interactions of the subunits. J. Biochem. **106**(3):423–429. DOI: 10.1093/oxfordjournals.jbchem.a122868.
60. Kaminaka, S., Takizawa, H., Handa, T., et al. (1992). Resonance Raman study on the active-site structure of a cooperative hemerythrin. Biochemistry **31**(30):6997–7002. DOI: 10.1021/bi00145a018.
61. Klotz, I.M., Klippenstein, G.L., and Hendrickson, W.A. (1976). Hemerythrin: Alternative Oxygen Carrier: Nature has developed an effective transport protein with a binuclear iron center in place of a heme. Science **192**(4237):335–344. DOI: 10.1126/science.1257769.
62. Bernhard, J.M. (1986). Characteristic assemblages and morphologies of benthic foraminifera from anoxic, organic-rich deposits; Jurassic through Holocene. J. Foram. Res. **16**(3):207–215. DOI: 10.2113/gsjfr.16.3.207.
63. Fenchel, T. (2014). Protozoa and Oxygen. Acta Protozool. **53**(1). DOI: 10.4467/16890027AP.13.0020.1117.
64. Hoffmann, F., Røy, H., Bayer, K., et al. (2008). Oxygen dynamics and transport in the Mediterranean sponge *Aplysina aerophoba*. Mar. Biol. **153**(6):1257–1264. DOI: 10.1007/s00227-008-0905-3.
65. Graham, J.B. (1988). Ecological and evolutionary aspects of integumentary respiration: body size, diffusion, and the Invertebrata. Am. Zool. **28**(3):1031–1045. DOI: 10.1093/icb/28.3.1031.
66. Manwell, C. (1960). Oxygen equilibrium of brachiopod *Lingula* hemerythrin. Science **132**(3426):550–551. DOI: 10.1126/science.132.3426.550.
67. Costa-Paiva, E.M., Schrago, C.G., and Halanych, K.M. (2017). Broad phylogenetic occurrence of the oxygen-binding hemerythrins in bilaterians. Genome Biol. Evol. **9**(10):2580–2591. DOI: 10.1093/gbe/evx181.
68. Gerdol, M., Luo, Y.-J., Satoh, N., and Pallavicini, A. (2018). Genetic and molecular basis of the immune system in the brachiopod *Lingula anatina*. Dev. Comp. Immunol. **82**:7–30. DOI: 10.1016/j.dci.2017.12.021.

69. Imai, K., Takizawa, H., Handa, T., and Kihara, H. (1991). Oxygen equilibrium characteristics of hemerythrins from the brachiopod, *Lingula unguis*, and the sipunculid, *Siphonosoma cumanense*. In Structure and Function of Invertebrate Oxygen Carriers, (Springer), pp. 179─189. 10.1007/978-1-4612-3174-5_24.

70. Richardson, D.E., Emad, M., Reem, R.C., and Solomon, E.I. (1987). Allosteric interactions in sipunculid and brachiopod hemerythrins. Biochemistry **26**(4):1003─1013. DOI: 10.1021/bi00378a005.

71. Martín-Durán, J.M., de Mendoza, A., Sebé-Pedrós, A., et al. (2013). A broad genomic survey reveals multiple origins and frequent losses in the evolution of respiratory hemerythrins and hemocyanins. Genome Biol. Evol. **5**(7):1435─1442. DOI: 10.1093/gbe/evt102.

72. Lieb, B., Altenhein, B., and Markl, J.r. (2000). The sequence of a gastropod hemocyanin (HtH1 from *Haliotis tuberculata*). J. Biol. Chem. **275**(8):5675─5681. DOI: 10.1074/jbc.275.8.5675.

73. Altenhein, B., Markl, J., and Lieb, B. (2002). Gene structure and hemocyanin isoform HtH2 from the mollusc *Haliotis tuberculata* indicate early and late intron hot spots. Gene **301**(1─2):53─60. DOI: 10.1016/S0378-1119(02)01081-8.

74. De Smet, L., Dimitrov, I., Debyser, G., et al. (2011). The cDNA sequence of three hemocyanin subunits from the garden snail *Helix lucorum*. Gene **487**(2):118─128. DOI: 10.1016/j.gene.2011.07.030.

75. Marxen, J.C., Pick, C., Oakley, T.H., and Burmester, T. (2014). Occurrence of hemocyanin in ostracod crustaceans. J. Mol. Evol. **79**(1):3─11. DOI: 10.1007/s00239-014-9636-x.

76. Miller, K.I., Cuff, M.E., Lang, W.F., et al. (1998). Sequence of the *Octopus dofleini* hemocyanin subunit: structural and evolutionary implications. J. Mol. Biol. **278**(4):827─842. DOI: 10.1006/jmbi.1998.1648.

77. Bergmann, S., Lieb, B., Ruth, P., and Markl, J. (2006). The hemocyanin from a living fossil, the cephalopod *Nautilus pompilius*: protein structure, gene organization, and evolution. J. Mol. Evol. **62**(3):362─374. DOI: 10.1007/s00239-005-0160-x.

78. Boisset, N., and Mouche, F. (2000). *Sepia officinalis* hemocyanin: a refined 3D structure from field emission gun cryoelectron microscopy. J. Mol. Biol. **296**(2):459─472. DOI: 10.1006/jmbi.1999.3460.

79. Morse, M.P., Meyhöfer, E., Otto, J.J., and Kuzirian, A.M. (1986). Hemocyanin respiratory pigment in bivalve mollusks. Science **231**(4743):1302–1304. DOI: 10.1126/science.3945826.

80. Terwilliger, N.B., Terwilliger, R.C., Meyhöfer, E., and Morse, M.P. (1988). Bivalve hemocyanins--a comparison with other molluscan hemocyanins. Comp. Biochem. Physiol. B Comp. Biochem. **89**(1):189–195. DOI: 10.1016/0305-0491(88)90282-9.

81. Bergmann, S., Markl, J., and Lieb, B. (2007). The first complete cDNA sequence of the hemocyanin from a bivalve, the protobranch *Nucula nucleus*. J. Mol. Evol. **64**(5):500–510. DOI: 10.1007/s00239-006-0036-8.

82. Kraus, D.W., and Colacino, J.M. (1986). Extended oxygen delivery from the nerve hemoglobin of *Tellina alternata* (Bivalvia). Science **232**(4746):90–92. DOI: 10.1126/science.232.4746.90.

83. Markl, J. (2013). Evolution of molluscan hemocyanin structures. Biochim. Biophys. Acta Proteins Proteomics **1834**(9):1840–1852. DOI: 10.1016/j.bbapap.2013.02.020.

84. Dewilde, S., Ebner, B., Vinck, E., et al. (2006). The nerve hemoglobin of the bivalve mollusc *Spisula solidissima*: molecular cloning, ligand binding studies, and phylogenetic analysis. J. Biol. Chem. **281**(9):5364–5372. DOI: 10.1074/jbc.M509486200.

85. Ullal, A.J., Litaker, R.W., and Noga, E.J. (2008). Antimicrobial peptides derived from hemoglobin are expressed in epithelium of channel catfish (*Ictalurus punctatus*, Rafinesque). Dev. Comp. Immunol. **32**(11):1301–1312. DOI: 10.1016/j.dci.2008.04.005.

86. Zhang, D.L., Guan, R.Z., Huang, W.S., and Xiong, J. (2013). Isolation and characterization of a novel antibacterial peptide derived from hemoglobin alpha in the liver of Japanese eel, *Anguilla japonica*. Fish Shellfish Immunol. **35**(3):625–631. DOI: 10.1016/j.fsi.2012.08.022.

87. Weber, R. (1982). Intraspecific adaptation of hemoglobin function in fish to oxygen availability. In Invited Lectures, (Elsevier), pp. 87–102. 10.1016/B978-0-08-027986-2.50014-1.

88. Manwell, C. (1966). Sea cucumber sibling species: polypeptide chain types and oxygen equilibrium of hemoglobin. Science **152**(3727):1393–1396. DOI: 10.1126/science.152.3727.1393.

89. Schillaci, D., Cusimano, M.G., Cunsolo, V., et al. (2013). Immune mediators of sea-cucumber *Holothuria tubulosa* (Echinodermata) as source of novel antimicrobial and anti-staphylococcal biofilm agents. Amb Express **3**(1):1–10. DOI: 10.1186/2191-0855-3-35.

90. Christensen, A.B., Herman, J.L., Elphick, M.R., et al. (2015). Phylogeny of echinoderm hemoglobins. PLoS ONE **10**(8):e0129668. DOI: 10.1371/journal.pone.0129668.
91. Christensen, A.B., and Colacino, J.M. (2000). Respiration in the burrowing brittlestar, *Hemipholis elongata* Say (Echinodermata, Ophiuroidea): a study of the effects of environmental variables on oxygen uptake. Comp. Biochem. Phys. A Mol. Integr. Phys. **127**(2):201─213. DOI: 10.1016/S1095-6433(00)00254-3.
92. Christensen, A.B., Colacino, J.M., and Bonaventura, C. (2003). Functional and biochemical properties of the hemoglobins of the burrowing brittle star *Hemipholis elongata* say (Echinodermata, Ophiuroidea). Biol. Bull. **205**(1):54─65. DOI: 10.2307/1543445.
93. Christensen, A.B. (2004). A new distribution record and notes on the biology of the brittle star *Ophiactis simplex* (Echinodermata: Ophiuroidea) in Texas. Tex. J. Sci. **56**(2):175─180. DOI.
94. Graham, J.B. (1988). Ecological and evolutionary aspects of integumentary respiration: body size, diffusion, and the invertebrata. Am. Zool. **28**(3):1031-1045. DOI: 10.1093/icb/28.3.1031.
95. Song, S., Starunov, V., Bailly, X., et al. (2020). Globins in the marine annelid *Platynereis dumerilii* shed new light on hemoglobin evolution in bilaterians. BMC Evol. Biol. **20**(1):165. DOI: 10.1186/s12862-020-01714-4.
96. Monahan-Earley, R., Dvorak, A.M., and Aird, W.C. (2013). Evolutionary origins of the blood vascular system and endothelium. J. Thromb. Haemost. **11**(s1):46-66. DOI: 10.1111/jth.12253.
97. Heim, N.A., Bakshi, S.H., Buu, L., et al. (2020). Respiratory medium and circulatory anatomy constrain size evolution in marine macrofauna. Paleobiology **46**(3):288-303. DOI: 10.1017/pab.2020.16.
98. Grice, K., Cao, C., Love, G.D., et al. (2005). Photic zone euxinia during the Permian-Triassic superanoxic event. Science **307**:706─709. DOI: 10.1126/science.1104323.
99. Cao, C., Love, G.D., Hays, L.E., et al. (2009). Biogeochemical evidence for euxinic oceans and ecological disturbance presaging the end-Permian mass extinction event. Earth Planet. Sci. Lett. **281**(3─4):188─201. DOI: 10.1016/j.epsl.2009.02.012.
100. Shen, Y., Farquhar, J., Zhang, H., et al. (2011). Multiple S-isotopic evidence for episodic shoaling of anoxic water during Later Permian mass extinction. Nat. Commun. **2**:210. DOI: 10.1038/ncomms1217.
101. Riccardi, A.L., Arthur, M.A., and Kump, L.R. (2006). Sulfur isotopic evidence for chemocline upward excursions during the end-Permian mass extinction. Geochim. Cosmochim. Acta

70(23):5740─5752. DOI: 10.1016/j.gca.2006.08.005.

102. Xie, S., Pancost, R.D., Huang, X., et al. (2007). Molecular and isotopic evidence for episodic environmental change across the Permo/Triassic boundary at Meishan in South China. Global Planet. Change **55**(1):56─65. DOI: 10.1016/j.gloplacha.2006.06.016.

103. Kaiho, K., Oba, M., Fukuda, Y., et al. (2012). Changes in depth-transect redox conditions spanning the end-Permian mass extinction and their impact on the marine extinction: Evidence from biomarkers and sulfur isotopes. Global Planet. Change **94─95**:20─32. DOI: 10.1016/j.gloplacha.2012.05.024.

104. Song, H., Wignall, P.B., Tong, J., et al. (2012). Geochemical evidence from bio-apatite for multiple oceanic anoxic events during Permian–Triassic transition and the link with end-Permian extinction and recovery. Earth Planet. Sci. Lett. **353─354**:12─21. DOI: 10.1016/j.epsl.2012.07.005.

105. Shen, W., Lin, Y., Xu, L., et al. (2007). Pyrite framboids in the Permian-Triassic boundary section at Meishan, China: Evidence for dysoxic deposition. Palaeogeogr., Palaeoclimatol., Palaeoecol. **253**(3─4):323─331. DOI: 10.1016/j.palaeo.2007.06.005.

106. Brennecka, G.A., Herrmann, A.D., Algeo, T.J., and Anbar, A.D. (2011). Rapid expansion of oceanic anoxia immediately before the end-Permian mass extinction. Proc. Natl. Acad. Sci. USA **108**(43):17631─17634. DOI: 10.1073/pnas.1106039108.

107. Lau, K.V., Maher, K., Altiner, D., et al. (2016). Marine anoxia and delayed Earth system recovery after the end-Permian extinction. Proc. Natl. Acad. Sci. USA **113**(9):2360─2365. DOI: 10.1073/pnas.1515080113.

108. Liao, W., Bond, D.P.G., Wang, Y., et al. (2017). An extensive anoxic event in the Triassic of the South China Block: A pyrite framboid study from Dajiang and its implications for the cause(s) of oxygen depletion. Palaeogeogr., Palaeoclimatol., Palaeoecol. **486**:86─95. DOI: 10.1016/j.palaeo.2016.11.012.

109. Liao, W., Wang, Y., Kershaw, S., et al. (2010). Shallow-marine dysoxia across the Permian-Triassic boundary: Evidence from pyrite framboids in the microbialite in South China. Sediment. Geol. **232**(1─2):77─83. DOI: 10.1016/j.sedgeo.2010.09.019.

110. Wang, L., Wignall, P.B., Wang, Y., et al. (2016). Depositional conditions and revised age of the Permo-Triassic microbialites at Gaohua section, Cili County (Hunan Province, South China).

Palaeogeogr., Palaeoclimatol., Palaeoecol. **443**:156─166. DOI: 10.1016/j.palaeo.2015.11.032.

111. Xiao, Y., Wu, K., Tian, L., et al. (2018). Framboidal pyrite evidence for persistent low oxygen levels in shallow-marine facies of the Nanpanjiang Basin during the Permian-Triassic transition. Palaeogeogr., Palaeoclimatol., Palaeoecol. **511**:243─255. DOI: 10.1016/j.palaeo.2018.08.012.

112. Zhou, W., Algeo, T.J., Ruan, X., et al. (2017). Expansion of photic-zone euxinia during the Permian–Triassic biotic crisis and its causes: Microbial biomarker records. Palaeogeogr., Palaeoclimatol., Palaeoecol. **474**:140─151. DOI: 10.1016/j.palaeo.2016.06.027.

113. Hinojosa, J.L., Brown, S.T., Chen, J., et al. (2012). Evidence for end-Permian ocean acidification from calcium isotopes in biogenic apatite. Geology **40**(8):743─746. DOI: 10.1130/g33048.1.

114. Payne, J.L., Turchyn, A.V., Paytan, A., et al. (2010). Calcium isotope constraints on the end-Permian mass extinction. Proc. Natl. Acad. Sci. USA **107**(19):8543─8548. DOI: 10.1073/pnas.0914065107.

115. Song, H., Song, H., Tong, J., et al. (2021). Conodont calcium isotopic evidence for multiple shelf acidification events during the early Triassic. Chem. Geol. **562**:120038. DOI: 10.1016/j.chemgeo.2020.120038.

116. Silva-Tamayo, J.C., Lau, K.V., Jost, A.B., et al. (2018). Global perturbation of the marine calcium cycle during the Permian-Triassic transition. Geol. Soc. Am. Bull. **130**(7─8):1323─1338. DOI: 10.1130/B31818.1.

117. Clarkson, M.O., Kasemann, S.A., Wood, R.A., et al. (2015). Ocean acidification and the Permo-Triassic mass extinction. Science **348**(6231):229─232. DOI: 10.1126/science.aaa0193.

118. Algeo, T.J., Shen, Y., Zhang, T., et al. (2008). Association of ^{34}S-depleted pyrite layers with negative carbonate δ^{13}C excursions at the Permian-Triassic boundary: Evidence for upwelling of sulfidic deep-ocean water masses. Geochem. Geophy. Geosy. **9**(4):Q04025. DOI: 10.1029/2007gc001823.

119. Algeo, T.J., Ellwood, B., Nguyen, T.K.T., et al. (2007). The Permian-Triassic boundary at Nhi Tao, Vietnam: Evidence for recurrent influx of sulfidic watermasses to a shallow-marine carbonate platform. Palaeogeogr., Palaeoclimatol., Palaeoecol. **252**(1─2):304─327. DOI: 10.1016/j.palaeo.2006.11.055.

120. Zhang, F., Algeo, T.J., Romaniello, S.J., et al. (2018). Congruent Permian-Triassic δ^{238}U records at Panthalassic and Tethyan sites: Confirmation of global-oceanic anoxia and validation of the U-

isotope paleoredox proxy. Geology **46**(4):327─330. DOI: 10.1130/g39695.1.

121. Wignall, P.B., and Hallam, A. (1992). Anoxia as a cause of the Permian/Triassic mass extinction: facies evidence from northern Italy and the western United States. Palaeogeogr., Palaeoclimatol., Palaeoecol. **93**(1─2):21─46. DOI: 10.1016/0031-0182(92)90182-5.

122. Newton, R.J., Pevitt, E.L., Wignall, P.B., and Bottrell, S.H. (2004). Large shifts in the isotopic composition of seawater sulphate across the Permo-Triassic boundary in northern Italy. Earth Planet. Sci. Lett. **218**(3─4):331─345. DOI: 10.1016/S0012-821X(03)00676-9.

123. Zhang, F., Shen, S.-z., Cui, Y., et al. (2020). Two distinct episodes of marine anoxia during the Permian-Triassic crisis evidenced by uranium isotopes in marine dolostones. Geochim. Cosmochim. Acta **287**:165─179. DOI: 10.1016/j.gca.2020.01.032.

124. Bond, D.P.G., and Wignall, P.B. (2010). Pyrite framboid study of marine Permian–Triassic boundary sections: A complex anoxic event and its relationship to contemporaneous mass extinction Geol. Soc. Am. Bull. **122**:1265─1279. DOI: 10.1130/B30042.1.

125. Clarkson, M., Wood, R., Poulton, S., et al. (2016). Dynamic anoxic ferruginous conditions during the end-Permian mass extinction and recovery. Nat. Commun. **7**:1223. DOI: 10.1038/ncomms1223.

126. Dolenec, T., Lojen, S., and Ramovs, A. (2001). The Permian-Triassic boundary in Western Slovenia (Idrijca Valley section): magnetostratigraphy, stable isotopes, and elemental variations. Chem. Geol. **175**:175─190. DOI: 10.1016/S0009-2541(00)00368-5.

127. Wignall, P.B., and Twitchett, R.J. (2002). Extent, duration, and nature of the Permian-Triassic superanoxic event. In Catastrophic events and mass extinctions; impacts and beyond: Geological Society of America Special Publication 356, C. Koeberl, and K.G. MacLeod, eds. pp. 395─413. 10.1130/SPE356.

128. Schneebeli-Hermann, E., Kürschner, W.M., Hochuli, P.A., et al. (2012). Palynofacies analysis of the Permian–Triassic transition in the Amb section (Salt Range, Pakistan): Implications for the anoxia on the South Tethyan Margin. J. Asian. Earth. Sci. **60**:225─234. DOI: 10.1016/j.jseaes.2012.09.005.

129. Wignall, P.B., and Newton, R. (2003). Contrasting deep-water records from the Upper Permian and Lower Triassic of South Tibet and British Columbia: evidence for a diachronous mass extinction. Palaios **18**(2):153─167. DOI: 10.1669/0883-1351(2003)18<153:CDRFTU>2.0.CO;2.

130. Schobben, M., Foster, W.J., Sleveland, A.R.N., et al. (2020). A nutrient control on marine anoxia during the end-Permian mass extinction. Nat. Geosci. **13**(9):640─646. DOI: 10.1038/s41561-020-0622-1.

131. Hays, L.E., Grice, K., Foster, C.B., and Summons, R.E. (2012). Biomarker and isotopic trends in a Permian–Triassic sedimentary section at Kap Stosch, Greenland. Org. Geochem. **43**:67─82. DOI: 10.1016/j.orggeochem.2011.10.010.

132. Hays, L.E., Beatty, T., Henderson, C.M., et al. (2007). Evidence for photic zone euxinia through the end-Permian mass extinction in the Panthalassic Ocean (Peace River Basin, Western Canada). Palaeoworld **16**(1─3):39─50. DOI: 10.1016/j.palwor.2007.05.008.
